# Exploring the associations of the triglyceride‒glucose index with thyroid function in subclinical hypothyroid patients: a cross-sectional study

**DOI:** 10.1186/s12944-025-02740-1

**Published:** 2025-10-21

**Authors:** Fawad Inayat, Sulaiman Shams, Noor Ullah, Arsalan Waqas Ahmad Shah, Ihtesham Ul Haq

**Affiliations:** 1 Department of Biochemistry, Abdul Wali Khan University, Mardan, 23200 Pakistan; 2 Khyber Medical University Institute of Paramedical Sciences, Peshawar, 25000 Pakistan; 3 Khyber Medical University Institute of Health Sciences, Swabi, 23340 Pakistan; 4 University of Engineering and Technology, Peshawar, 25000 Pakistan

**Keywords:** *Triglycerides*, *Glucose*, *Insulin resistance*, *Thyroid gland/Physiopathology*, *Hypothyroidism*, *Subclinical*

## Abstract

**Background:**

Subclinical hypothyroidism (SCH) often occurs in association with the emergence of the metabolic disorder like insulin resistance (IR). This study aimed to determine the relationship between the Triglyceride Glucose (TyG) index and thyroid function in patients with subclinical hypothyroidism (SCH), to identify metabolic predictors of thyroid dysfunction.

**Methods:**

This cross-sectional study used convenience sampling, and data were collected after written informed consent. This study was conducted at tertiary care hospitals in Peshawar, Pakistan, and included 2024 subclinical hypothyroid patients with an age > 19 years. Individuals with any thyroid condition, diabetes, cardiovascular disorders or chronic liver conditions were excluded. Regression, ANOVA, and long short-term memory (LSTM) models were used to predict the TyG index, TSH, T3, and FT4 levels. All analyses were performed using R version 4.3.0 and Python. The result was considered statistically significant with *P* < 0.05.

**Results:**

The male-to-female ratio was 1:2, and the highest group included 41 50-year-olds (40.3%). Regression analysis revealed an inverse association between the TyG index and T3 level (*β* = -0.313, *P* < 0.0001) and a positive association with HbA1c (*β* = 0.198; *P* < 0.0001), indicating a relationship between a higher TyG index and IR and poor glycemic control. The values of HDL were negatively correlated with the TyG index (*β* = -0.221, *P* < 0.0001); conversely, LDL was positively correlated to TyG (*β* = 0.234, *P* < 0.0001). The LSTM model presented high predictive accuracy with small mean squared errors, 0.00034 for the TyG index, 0.0015 for T3, and 0.0113 for T4.

**Conclusion:**

The findings of this study demonstrated that the TyG index can be an effective and important parameter of metabolic health and a predictor of thyroid function in subclinical hypothyroid patients. These findings underscore the importance of early identification of metabolic risk factors for thyroid dysfunction, which can contribute to improved health outcomes and reduce the long-term burden of endocrine and cardiovascular diseases at the population level. Moreover, the results of the current research cannot be generalized, as it is a cross-sectional study.

**Supplementary Information:**

The online version contains supplementary material available at 10.1186/s12944-025-02740-1.

## Introduction

Insulin resistance (IR) is a condition in which the body becomes resistant to the effects of insulin. It is also a detrimental predisposing risk factor for a wide range of metabolic complications, including type 2 diabetes mellitus (T2DM), cardiovascular disease (CVD), polycystic ovary syndrome (PCOS), and dyslipidemia [1]. The hyper-insulinaemic-euglycaemic clamp (HEC) is the gold-standard of measuring IR. The complexity, expense and time of using this method have restricted its use in the clinic and research setting [2, 3]. In addition, pseudo-indices such as the homeostatic model assessment of insulin resistance (HOMA-IR), metabolic score of insulin resistance (METS-IR), quantitative insulin sensitivity index (QUICKI) and Matsuda index are also simulated [4]. Among these indices, the triglyceride‒glucose (TyG) index, employs only two parameters, i.e., fasting plasma glucose (FPG) and triglyceride (TG) concentrations and has gained increased attention because of its simplicity and applicability as a potential predictor of IR and other metabolic disorders [5, 6].

The inconsistency in many metabolic processes is associated with the thyroid hormone (THs) and glucose metabolism [7]. Hyperthyroidism is caused by excess synthesis and secretion of THs and is characterised by irregular insulin signalling [8]. Moreover, hypothyroidism is directly linked with metabolic disorders, such as IR [8, 9]. SCH is characterized by elevated thyroid-stimulating hormone (TSH) levels and normal free thyroxine (FT4) levels. Generally, subclinical hypothyroidism is defined as TSH > 4.0 mIU/L, depending on which country the patient is screened in and which is the National Institute for Health and Care Excellence (NICE) [10], Although similar thresholds are used by the European Thyroid Association (ETA) and American Thyroid Association (ATA), in general, TSH levels between 4.0 and 10.0 mIU/L are commonly regarded as mild SCH [11, 12]. Persistent elevation should be confirmed over time, and treatment is generally recommended for individuals with a TSH level greater than 10 mIU/L or for symptomatic individuals. However, studies examining the relationship between thyroid function and IR have produced conflicting results, and there is limited research on the associations between the TyG index and thyroid function in patients with SCH [13, 14].

The present study was undertaken to test the hypothesis that the TyG index is significantly correlated with thyroid function in patients with SCH. While previous studies have investigated insulin resistance and metabolic disturbances in thyroid disorder patients, there is limited evidence about the presence of TyG index as a surrogate index in this population. This study adds to the existing knowledge by being among the first to systematically evaluate the relationship between the TyG index and TH in SCH patients and by employing both regression models and LSTM, thereby highlighting the potential utility of the TyG index as a novel indicator of metabolic risk and thyroid-related complications.

## Materials and methods

### Study settings and design

This observational cross-sectional study was conducted in three tertiary care hospitals namely Hayatabad Medical Complex (HMC), Khyber Teaching Hospital (KTH), and Lady Reading Hospital (LRH) in urban area of Peshawar, Pakistan from January 2022 to August 2022. The sampling technique was easily determined and conceptualised to introduce the participants to the study information, orally. Written informed consent was received to take part in the study.

### Inclusion criteria

- Adults aged 18–65 years.
- SCH participants were indicated as having high TSH and normal FT4.
- Individuals who have not been previously diagnosed with DM, CVD, cerebrovascular diseases, malignant conditions or any chronic liver conditions.

### Exclusion criteria

- Women who are pregnant.
- Patients with confirmed thyroid disorders other than SCH.
- Patients with or without lipid-lowering therapy.

### Sample size

The final sample size (2024) was calculated using the expected prevalence of SCH in the target population, as it was reported to be 4–10% among a general adult population [15, 16]. This estimation ensured the sufficiency of statistical power (100%) to achieve clinically significant estimations of the primary outcomes, which included the parameters of thyroid function and metabolic indicators, in this case, at a significance level of *P* = 0.05. There is reliance on prior effect sizes in the discussion of the power observed within comparable populations, and this reliance extends to diagnostic criteria, as noted in its inclusion criteria [17, 18].

### Demographic, lifestyle, and anthropometric parameters

Data including demographic information (age, sex, height, weight and BMI) were collected [19]. BMI was computed as the ratio of kg to height squared (m^2^) [20].

### Measurement of laboratory indices

Sampling of the blood was done in the morning and a minimum of 8 h fasting was observed prior to the blood being collected. The gel tube was filled with five ml of blood into a 22-gauge needle/5 ml syringe into which blood was collected. An FT4 and TSH serum was analysed using a C501 analyser (Roche Diagnostics). These analyses were conducted through the concept of electrochemiluminescence immunoassays (ECLIAs). Blood FPG and HbA1c were tested. The HgA1c was determined using High-Performance liquid Chromatography (HPLC) method. A Cobas 6000 Roche diagnostics analyser was used to validate it. TyG index was computed using the natural logarithm of fasting triglycerides and multiplying it with the natural logarithm of fasting glucose and dividing the two logarithms by 2. ln TyG = ln (fasting triglyceride (mg/dl)fasting glucose (mg/dl)/2 [21, 22]. The total serum cholesterol, TG, high-density lipoprotein (HDL), low-density lipoprotein (LDL), and FPG levels of the participants were also determined.

### Statistical analysis

The mean difference among groups of continuous variables was determined using ANOVA, and the chi-square test was used for categorical variables. Multivariate linear regression was utilized to study the continuous outcomes and was adjusted for confounding variables and quantify the associations. The problem of multicollinearity of predictors (TyG index, sex, age, BMI) has also been evaluated as a variance inflation factor (VIF). The VIFs of all the variables were considerably lower (< 2) than the recommended cut-off of 5, suggesting a lack of multicollinearity. The regression of the TyG index with thyroid function was performed via a multivariable linear regression model, and all the regulative-related requirements to regress, including normality, were assumed to be met. In the present study, an LSTM model that uses input one-time series data (sequence one), an LSTM layer with 64 cell units, a 0.2 dropout ratio, and one-unit output was used. This was accomplished through progressive, progressive and emergent theoretical learning models, that is, through Adam optimization (learning rate = 0.001) and loss, the mean squared error (MSE). The data were divided into training and test sets at proportions of 80–20, and 5-fold cross-validation was performed to ensure that the model used was not overtrained. The analyses were performed via R and Python, with *P* < 0.05 regarded as the significance level.

### Ethical considerations

The research was approved by the Research Ethical Committee (REC) at Iqra National University, Peshawar, Pakistan (No. INU/AHS/57 − 22). Written informed consent was obtained from all participants prior to data collection.

## Results

### Baseline characteristics of the participants

The demographic baseline description of the study population provided some critical aspects. The study comprised 2024 participants, and a larger portion of them are female (66.9%). The age group 41–50 years is the prominent one (40.3%), followed by the 51–60 years (27.5%). The mean BMI (25.09 ± 1.12) of the participants is indicative of overweight. Thyroid function tests revealed that the mean level of TSH was slightly greater (4.77 ± 0.38). The Lipid profile indicated elevated Cholesterol and LDL compared with HDL, which is Likely a risk factor for cardiovascular issues. The TyG index mean value in the study population is 4.80, which exceeds the standard cut-off point (4.67) identified by Simental-Mendia et al. (2008) [22]. The liver function tests (alanine aminotransferase (ALT), alkaline phosphatase (ALP), aspartate aminotransferase (AST), and gamma-glutamyl transferase (GGT)) and Complete Blood Count (CBC) were normal (Table 1).

**Table 1:** Baseline characteristics of the study participants

Characteristics	Values
Sex (%)	
Male	670 (33.1%)
Female	1354 (66.9%)
Age Group (Years)	
21–30 Years	128 (6.3%)
31–40 Years	376 (18.6%)
41–50 Years	815 (40.3%)
51–60 Years	556 (27.5%)
61–70 Years	149 (7.4%)
Height (Feet)	5.44 ± 0.244
Weight (Pounds)	148.63 ± 10.74
Body Mass Index (BMI)	25.09 ± 1.12
TG	163.91 ± 17.33
TSH (Milliunit/Liter)	4.77 ± 0.38
FT4 (ng/dl)	0.99 ± 0.097
T3 (pg/ml)	1.67 ± 0.44
Cholesterol (mg/dl)	256.38 ± 49.18
LDL (mg/dl)	130.093 ± 27.54
HDL (mg/dl)	42.82 ± 13.96
HbA1c	6.59 ± 0.50
FPG (mg/dl)	90.80 ± 9.69
TyG Index	4.80 ± 0.069
ALT (U/L)	39.72 ± 27.71
ALP (U/L)	126.63 ± 60.76
AST (U/L)	46.40 ± 18.73
GGT (U/L)	58.35 ± 17.05
RBC (million cells/µL)	4.27 ± 0.85
WBC (thousand cells/µL)	4.44 ± 0.73

### Gender differences in the metabolic and thyroid profiles of subclinical hypothyroid patients

This study compared demographic, lipid, thyroid, liver, and hematological parameters. The average weights and heights of the males (161.09 ± 9.39 pounds and 5.71 ± 0.20 feet, respectively) were greater than females (142.47 ± 3.76 pounds and 5.31 ± 0.13 feet, respectively; *P* < 0.001). The female exhibited higher FPG (92.21 ± 10.91 mg/dl, *P* < 0.001), cholesterol (262.66 ± 44.56 mg/dl; *P* < 0.001), HDL (44.84 ± 14.25 mg/dl; *P* < 0.001), LDL (133.54 ± 25.40 mg/dl; *P* < 0.001), and TSH (4.85 ± 0.28 milliunits/L; *P* < 0.001) compared to males. All these differences in metabolic and thyroid profiles are due to hormones: estrogens in women stimulate lipid metabolic activities and inhibit thyroid-binding proteins, while testosterone in men stimulates the metabolic rate and hepatic enzyme synthesis. However, the finding revealed that the males have higher concentration of T3 (2.05 ± 0.32 pg/ml; *P* < 0.001), ALT (49.59 ± 31.34 U/L; *P* < 0.001), GGT (65.07 ± 15.72 U/L; *P* < 0.001), RBC counts (4.65 ± 0.65 million cells/µl; *P* < 0.001) and WBC counts (4.53 ± 0.61 thousand cells/µl; *P* < 0.01) than females. The TyG index did not differ significantly between sexes (*P* > 0.05) (Table 2).

**Table 2:** Gender-based comparison of metabolic, thyroid, and haematological parameters among subclinical hypothyroid patients

Parameter	Male: Mean (SD)	Female: Mean (SD)	Levene’s Statistic	*P* value
BMI (kg/m²)	24.83 (1.05)	25.23 (1.13)	3.43	0.064
Weight (pounds)	161.09 (9.39)	142.47 (3.76)	493.68	< 0.001*
Height (feet)	5.71 (0.20)	5.31 (0.13)	277.16	< 0.001*
FPG (mg/dl)	87.93 (5.53)	92.21 (10.91)	152.85	< 0.001*
HbA1c (%)	6.59 (0.49)	6.59 (0.50)	1.26	0.262
TG (mg/dl)	159.57 (19.81)	166.05 (15.53)	56.34	< 0.001*
Cholesterol (mg/dl)	243.70 (55.29)	262.66 (44.56)	171.24	< 0 0.001*
TSH (milliunit/L)	4.61 (0.49)	4.85 (0.28)	198.52	< 0 0.001*
T3 (pg/ml)	2.05 (0.32)	1.48 (0.37)	39.75	< 0.001*
FT4 (ng/dl)	1.01 (0.14)	0.98 (0.06)	86.1	< 0.001*
TyG Index (unitless)	4.77 (0.07)	4.81 (0.07)	1.83	0.176
HDL (mg/dl)	38.72 (12.41)	44.84 (14.25)	35.64	< 0.001*
LDL (mg/dl)	123.12 (30.27)	133.54 (25.40)	38.69	< 0.001*
ALT (U/L)	49.59 (31.34)	34.84 (24.30)	133.42	< 0.001*
ALP (U/L)	113.19 (54.72)	133.28 (62.50)	86.35	< 0.001*
GGT (U/L)	65.07 (15.72)	55.03 (16.71)	6.62	0.01*
WBC (thousand cells/µL)	4.53 (0.61)	4.39 (0.78)	10.01	0.002*
RBC (million cells/µL)	4.65 (0.65)	4.08 (0.87)	263.27	< 0.001*

*A *P* value less than 0.05 was considered statistically significant

### Age-related variations in the metabolic, thyroid, and haematological profiles of subclinical hypothyroid patients

Age-wise, the analysis revealed significant changes in metabolic, thyroid, and haematological data. BMI and weight decrease with increasing age, and younger patients (21–30 years) had the highest BMI (25.46 ± 1.11 kg/m^2^; *P* < 0.001) and weight (*P* < 0.001) compared with older patients (61–70 years). The Height gain with age was slight, particularly beyond the ages of 51–60 years and 61–70 years, and highly variable (*P* < 0.001). The FPG reached a maximum in groups 31–40 (95.05 ± 12.74 mg/dl; *P* < 0.001) and decreased significantly in the older age group. Similarly, the maximum HbA1c was observed in the middle-aged group (31–40 years), whereas the lowest level was found in the younger and older groups (*P* < 0.001). TG levels significantly increased with age (168.49 ± 21.93 mg/dl; *P* < 0.001), suggesting that lipid metabolism becomes more complex with increasing age. The cholesterol levels, however, exhibited very different trends, with the youngest age group having the highest cholesterol levels and decreases in old age group (*P* < 0.001), while the TSH remained the same among the age groups (*P* = 0.568). However, the mean T3 and FT4 values are significantly different, whereas T3 was found to be higher at the youngest age, and FT4 was unstable with age (*P* < 0.001). Minor but notable variations were observed in the TyG index across the age group (*P* < 0.001). The HDL level increases, and LDL decreases with an increase in age (*P* < 0.001), which implies that there might be an age-related difference in the level of lipids. The changes in the levels of liver enzymes (ALT, ALP, GGT) and hematological parameters (WBC, RBC) also differed in terms of age (*P* < 0.001) (Table 3).

**Table 3:** Age-based differences in metabolic, thyroid, and hematological parameters among subclinical hypothyroid patients

Parameter	21–30: Mean (SD)	31–40: Mean (SD)	41–50: Mean (SD)	51–60: Mean (SD)	61–70: Mean (SD)	F-statistic	*P* value
BMI (kg/m²)	25.46 (1.11)	25.12 (1.08)	25.19 (1.24)	25.00 (0.95)	24.61 (0.90)	13.29	< 0.001*
Weight (pounds)	150.75 (12.32)	147.02 (10.36)	148.08 (9.67)	149.82 (11.12)	149.49 (13.37)	5.9	< 0.001*
Height (feet)	5.44 (0.32)	5.40 (0.23)	5.43 (0.23)	5.47 (0.24)	5.48 (0.27)	6.39	< 0.001*
Fasting Sugar Level (mg/dl)	91.51 (8.10)	95.05 (12.74)	89.80 (9.41)	89.95 (7.63)	88.02 (6.90)	25.7	< 0.001*
HbA1c (%)	6.24 (0.33)	6.62 (0.49)	6.68 (0.50)	6.59 (0.49)	6.35 (0.43)	33.99	< 0.001*
Triglyceride (mg/dl)	152.98 (18.83)	159.76 (14.09)	165.60 (16.65)	165.52 (17.12)	168.49 (21.93)	25	< 0.001*
Cholesterol (mg/dl)	273.75 (30.76)	254.26 (44.37)	257.90 (48.50)	257.11 (52.58)	235.79 (56.92)	11.14	< 0.001*
TSH (milliunit/L)	4.76 (0.43)	4.75 (0.39)	4.78 (0.38)	4.77 (0.37)	4.81 (0.40)	0.74	0.568
T3 (pg/ml)	1.87 (0.36)	1.55 (0.50)	1.69 (0.45)	1.67 (0.40)	1.69 (0.38)	14.41	< 0.001*
FT4 (ng/dl)	0.97 (0.04)	0.99 (0.10)	0.97 (0.05)	1.03 (0.14)	0.94 (0.05)	41.9	< 0.001*
Triglyceride Glucose Index (unitless)	4.77 (0.06)	4.81 (0.07)	4.80 (0.07)	4.80 (0.07)	4.80 (0.08)	7.78	< 0.001*
HDL (mg/dl)	43.59 (7.47)	39.52 (9.17)	40.58 (12.49)	44.83 (16.17)	55.19 (18.45)	46.57	< 0.001*
LDL (mg/dl)	141.51 (8.96)	134.42 (23.32)	130.03 (27.39)	128.72 (30.87)	114.87 (28.60)	20.3	< 0.001*
ALT (U/L)	45.70 (26.32)	35.52 (30.63)	45.74 (27.61)	34.45 (24.15)	31.96 (26.60)	22.09	< 0.001*
ALP (U/L)	173.10 (55.44)	117.80 (60.93)	128.60 (55.01)	116.14 (60.47)	137.34 (73.86)	27.6	< 0.001*
GGT (U/L)	69.48 (10.77)	55.01 (17.58)	58.76 (16.28)	57.06 (17.85)	59.79 (17.14)	19.09	< 0.001*
WBC (thousand cells/µL)	4.41 (0.52)	4.91 (0.93)	4.25 (0.66)	4.37 (0.61)	4.59 (0.54)	62.23	< 0.001*
RBC (million cells/µL)	4.33 (0.80)	4.66 (0.67)	4.13 (0.90)	4.21 (0.83)	4.19 (0.77)	28.35	< 0.001*

A *P* value less than 0.05 was considered statistically significant

### Correlation analysis of metabolic, thyroid, and biochemical parameters in subclinical hypothyroid patients

Spearman correlation showed that the associations of metabolite with thyroid and biochemical parameters were significant in SCH patients. The TyG was well correlated with FPG (*r* = 0.635, *P* < 0.001) and moderately correlated with HbA1c (*r* = 0.220, *P* < 0.001). The cholesterol value was significantly correlated with LDL (*r* = 0.856, *P* < 0.001) and negatively with HDL (*r* = −0.544, *P* < 0.001) which reflects a desirable lipid pattern in the patient with subclinical hypothyroidism. The concentration of cholesterol correlated positively with TSH (*r* = 0.303, *P* < 0.001), suggesting that there is a reciprocal relation between cholesterol and TSH. TSH positively correlated with HbA1c (*r* = 0.286, *P* < 0.001) and negatively correlated with BMI (*r* = −0.354, *P* < 0.001), which may be related to poor glycemia and low BMI in these patients. T3 was negatively correlated with cholesterol (*r* = −0.189, *P* < 0.001) and positively correlated with ALT (*r* = 0.287, *P* < 0.001), which suggested some complicated relationships between TH, lipoprotein metabolism and liver enzymes (Table 4).

**Table 4:** Spearman correlation analysis of metabolic, thyroid, and biochemical parameters in subclinical hypothyroid patients

Parameter	Details	TG	HbA1c	FPG	TyG index	T3	FT4	TSH	Chole sterol	BMI	Weight	LDL	HDL	ALT	ALP	AST
TG (mg/dl)	P.C	1	0.058**	−0.132**	0.678**	−0.168**	−0.033**	0.078**	0.241**	0.071**	−0.176**	0.086**	−0.088**	−0.033	− 0.053*	−0.076**
	Sig.		0.009	0.0001	0.0001	0.0001	0.0001	0.0001	0.0001	0.001	0.0001	0.0001	0.0001	0.138	0.018	0.001
HbA1c	P.C		1	0.224**	0.220**	−0.262	0.094**	0.130**	0.286**	0.284**	−0.015	0.270**	−0.223	−0.123**	−0.301**	−0.165**
	Sig.			0.0001	0.0001	0.0001	0.0001	0.0001	0.0001	0.0001	0.497	0.0001	0.0001	0.0001	0.0001	0.0001
FPG (mg/dl)	P.C			1	0.635**	−0.458**	−0.010	−0.002	0.146**	0.188**	−0.109**	0.244**	−0.103**	−0.025	0.088**	−0.219**
	Sig.				0.0001	0.0001	0.639	0.932	0.0001	0.0001	0.0001	0.0001	0.0001	0.269	0.0001	0.0001
TyG index	P.C				1	0.472**	− 0.020	0.063**	0.306**	0.187**	− 0.226**	0.257**	− 0.150**	− 0.054*	0.020	− 0.219**
	Sig.					0.0001	0.372	0.005	0.0001	0.0001	0.0001	0.0001	0.0001	0.016	0.377	0.0001
T3 (pg/ml)	P.C					1	0.159**	−0.003	−0.189**	−0.191**	0.433**	−0.266**	0.002	0.179**	0.092**	0.287**
	Sig.						0.0001	0.907	0.0001	0.0001	0.0001	0.0001	0.916	0.0001	0.0001	0.0001
FT4 (ng/ml)	P.C						1	0.096**	−0.006	−0.035	−0.027	0.005	−0.026	0.016	−0.023	−0.002
	Sig.							0.0001	0.796	0.111	0.220	0.822	0.247	0.464	0.311	0.945
TSH (milliunit/L)	P.C							1	0.303**	0.060**	−0.354**	0.171**	−0.042	−0.081**	0.093**	−0.053**
	Sig.								0.0001	0.007	0.0001	0.0001	0.062	0.0001	0.0001	0.010
Cholesterol (mg/dl)	P.C								1	0.136**	−0.169**	0.856**	−0.544**	−0.294**	0.026	0.104**
	Sig.									0.0001	0.0001	0.0001	0.0001	0.0001	0.283	0.0001
BMI	P.C									1	−0.141**	0.079**	−0.163**	0.144**	−0.089**	−0.062**
	Sig.										0.0001	0.0001	0.0001	0.0001	0.0001	0.006
Weight (Pounds)	P.C										1	−0.058**	−0.108**	0.101**	−0.018	0.015
	Sig.											0.0001	0.0001	0.0001	0.421	0.512
LDL (mg/dl)	P.C											1	−0.566**	−0.391**	0.077**	0.053*
	Sig.												0.0001	0.0001	0.0001	0.018
HDL (mg/dl)	P.C												1	0.059**	0.049*	0.047*
	Sig.													0.008	0.026	0.036
ALT (U/L)	P.C													1	0.227**	−0.050*
	Sig.														0.0001	0.023
ALP (U/L)	P.C														1	0.490**
	Sig.															0.0001

*PC *Pearson Correlation*, Sig *Significance

**A *P* value less than 0.001 was considered statistically significant

### Regression analysis of the TyG index, sex, age, and BMI on various metabolic and thyroid parameters

The regression model revealed that the associations between the TyG index and sex, age, BMI, and other metabolic and thyroid parameters in subclinical hypothyroid patients were strong. The major predictors of FT4 included female sex, whose levels were lower (*β* = −0.150, *P* < 0.0001), and the TyG index; age and BMI were not notable predictors. T3 levels also showed a significant inverse correlation with the TyG index (B = −0.313; *P* < 0.0001). Males have an insignificant association with T3 (*β* = −0.501, *P* < 0.0001), with a negative correlation with BMI. TSH was a strong positive predictor since females had higher values (*β* = 0.314, *P* < 0.0001). The HbA1c was positively associated with the TyG index (*β* = 0.198, *P* < 0.0001), indicating that a higher TyG index would imply worsening glycemic control. The LDL (*β* = 0.234, *P* < 0.0001), and HDL (*β*=−0.221, *P* < 0.0001) were significantly associated with the TyG index. In case of cholesterol, the TyG index is a strong positive predictor (*β* = 0.273, *P* < 0.0001) for males and younger participants. The female had an inverse association with the ALT (*β* = −0.285, *P* < 0.0001), age and BMI (Table 5).

**Table 5:** Regression analysis of the effects of the TyG index, sex, age, and BMI on various metabolic and thyroid parameters in subclinical hypothyroid patients

Variable	Parameters	B	Β	SE	T	*R* Square	Tolerance (VIF)	*P* Value
FT4	TyG index	0.035	0.025	0.033	1.078	0.000	0.888 (1.126)	0.281
	Gender (Male)	−0.031	−0.150	0.005	−6.44	0.021	0.897 (1.115)	< 0.0001*
	Age	0.004	0.039	0.002	1.741	0.002	0.976 (1.024)	0.082
	BMI	−0.001	−0.010	0.002	−0.439	0.001	0.934 (1.071))	0.660
T3	TyG index	−1.993	−0.313	0.11	−18.001	0.223	0.888 (1.126)	< 0.0001*
	Gender	−0.471	−0.501	0.016	−29.00	0.361	0.897 (1.115)	< 0.0001*
	Age	−0.012	−0.028	0.007	−1.675	0.000	0.976 (1.024)	0.094
	BMI	−0.021	−0.052	0.007	−3.098	0.036	0.934 (1.071))	0.002*
TSH	TyG index	−0.195	−0.035	0.124	−1.576	0.004	0.888 (1.126)	0.115
	Gender	0.255	0.314	0.018	14.06	0.093	0.897 (1.115)	< 0.0001*
	Age	0.019	0.048	0.008	2.264	0.001	0.976 (1.024)	0.024
	BMI	0.007	0.021	0.007	0.949	0.004	0.934 (1.071))	0.343
HBA1C	TyG index	1.410	0.198	0.158	8.917	0.048	0.888 (1.126)	< 0.0001*
	Gender	−0.102	−0.097	0.023	−4.377	0.000	0.897 (1.115)	< 0.0001*
	Age	0.016	0.032	0.010	1.525	0.000	0.976 (1.024)	0.127
	BMI	0.118	0.267	0.010	12.358	0.081	0.934 (1.071))	< 0.0001*
LDL	TyG index	92.675	0.234	8.842	10.482	0.066	0.888 (1.126)	< 0.0001*
	Gender	5.830	0.100	1.298	4.492	0.032	0.897 (1.115)	< 0.0001*
	Age	−4.951	−0.179	0.587	−8.439	0.031	0.976 (1.024)	< 0.0001*
	BMI	−0.132	−0.005	0.535	−0.247	0.006	0.934 (1.071))	0.805
HDL	TyG index	−44.482	−0.221	4.341	−10.247	0.023	0.888 (1.126)	< 0.0001*
	Gender	9.130	0.308	0.637	14.329	0.043	0.897 (1.115)	< 0.0001*
	Age	2.968	0.212	0.288	10.305	0.043	0.976 (1.024)	< 0.0001*
	BMI	−1.798	−0.144	0.263	−6.842	0.026	0.934 (1.071))	< 0.0001*
Cholesterol	TyG index	193.147	0.273	15.731	12.278	0.094	0.888 (1.126)	< 0.0001*
	Gender	8.975	0.086	2.309	3.887	0.033	0.897 (1.115)	< 0.0001*
	Age	−4.442	−0.090	1.044	−4.256	0.009	0.976 (1.024)	< 0.0001*
	BMI	2.563	0.058	0.952	2.691	0.018	0.934 (1.071))	0.007*
ALT	TyG index	−0.091	0.00	8.917	−0.010	0.003	0.888 (1.126)	0.992
	Gender	−16.788	−0.285	1.309	−12.827	0.063	0.897 (1.115)	< 0.0001*
	Age	−2.354	−0.085	0.592	−3.978	0.009	0.976 (1.024)	< 0.0001*
	BMI	4.454	0.180	0.540	8.252	0.021	0.934 (1.071))	< 0.0001*
ALP	TyG index	−3.719	−0.004	20.172	−0.184	0.000	0.888 (1.126)	0.854
	Gender	22.322	0.173	2.961	7.539	0.024	0.897 (1.115)	< 0.0001*
	Age	−6.024	−0.099	1.338	−4.500	0.008	0.976 (1.024)	< 0.0001*
	BMI	−7.051	−0.130	1.221	−5.775	0.008	0.934 (1.071))	< 0.0001*

*A *P* value less than 0.05 was considered statistically significant

### LSTM model predictions for TyG index, TSH, T3, and T4 levels

**Fig. 1:**
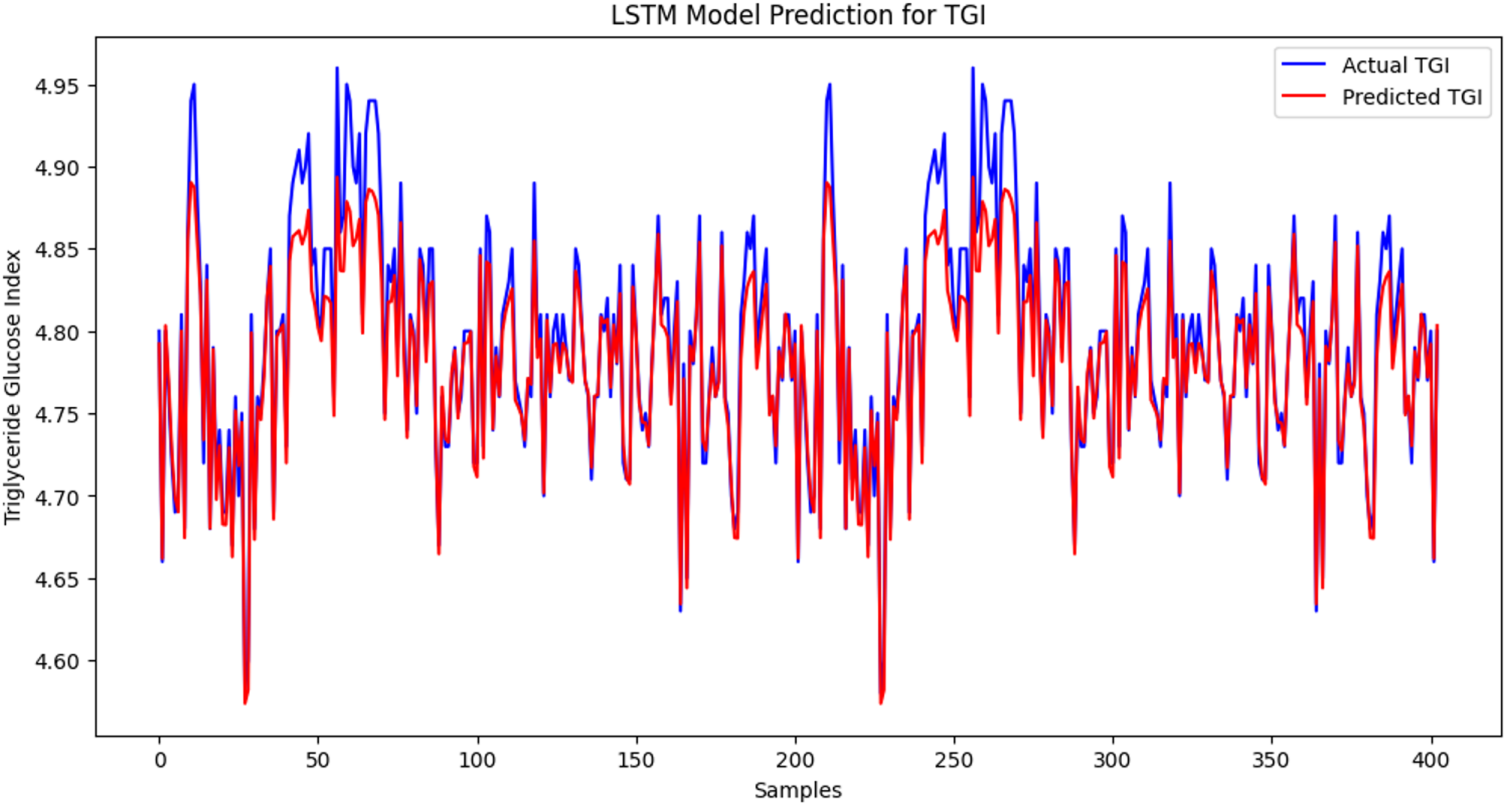
LSTM model prediction for the TyG index: A comparison of the actual (blue line) and LSTM-predicted (red line) values of the TyG index demonstrated high predictive accuracy (MSE = 0.00034) in subclinical hypothyroid patients. The vertical x-axis represents the standardized TyG index values, and the horizontal axis represents a sequential index of the data samples on the x-axis (not actual time). A subgroup of 400 samples is represented to help make it clear, which enables one to capture the overall tendency and all minor differences in the TyG index. The small differences between the actual and predicted values reveal that the model indeed can track this relevant metabolic index

**Fig. 2:**
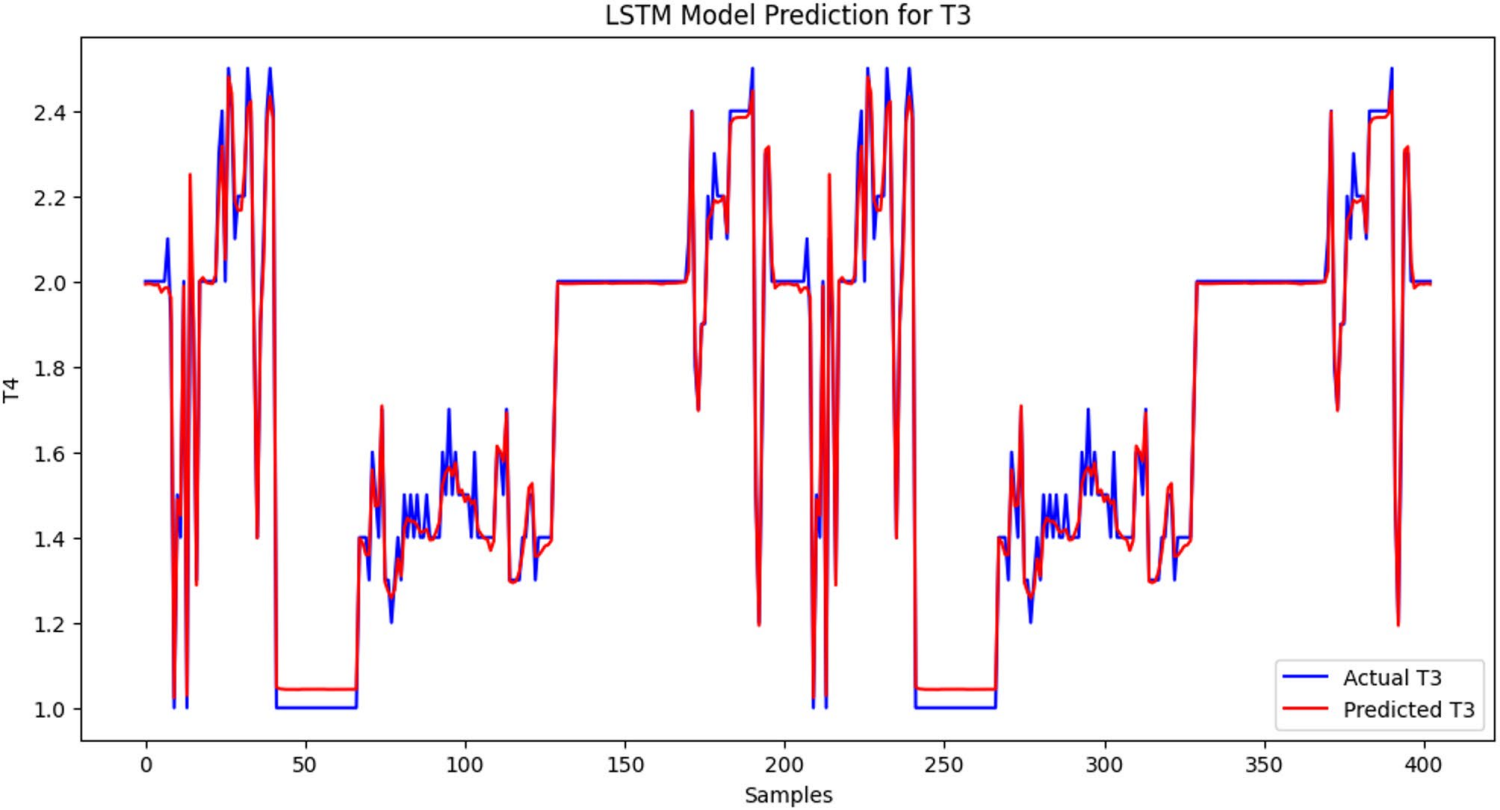
LSTM model prediction for T3 levels: The cross-validation results based on time recorded high predictability between real (blue line) and LSTM-predicted (red line) T3 hormone levels in patients with subclinical hypothyroidism (MSE = 0.0015). Among the peculiarities, it is necessary to say that the model can help identify not only a gradual trend but also the process that suggests a sharp alteration in T3 levels, which confirms its effectiveness as a monitoring mechanism of thyroid function

**Fig. 3:**
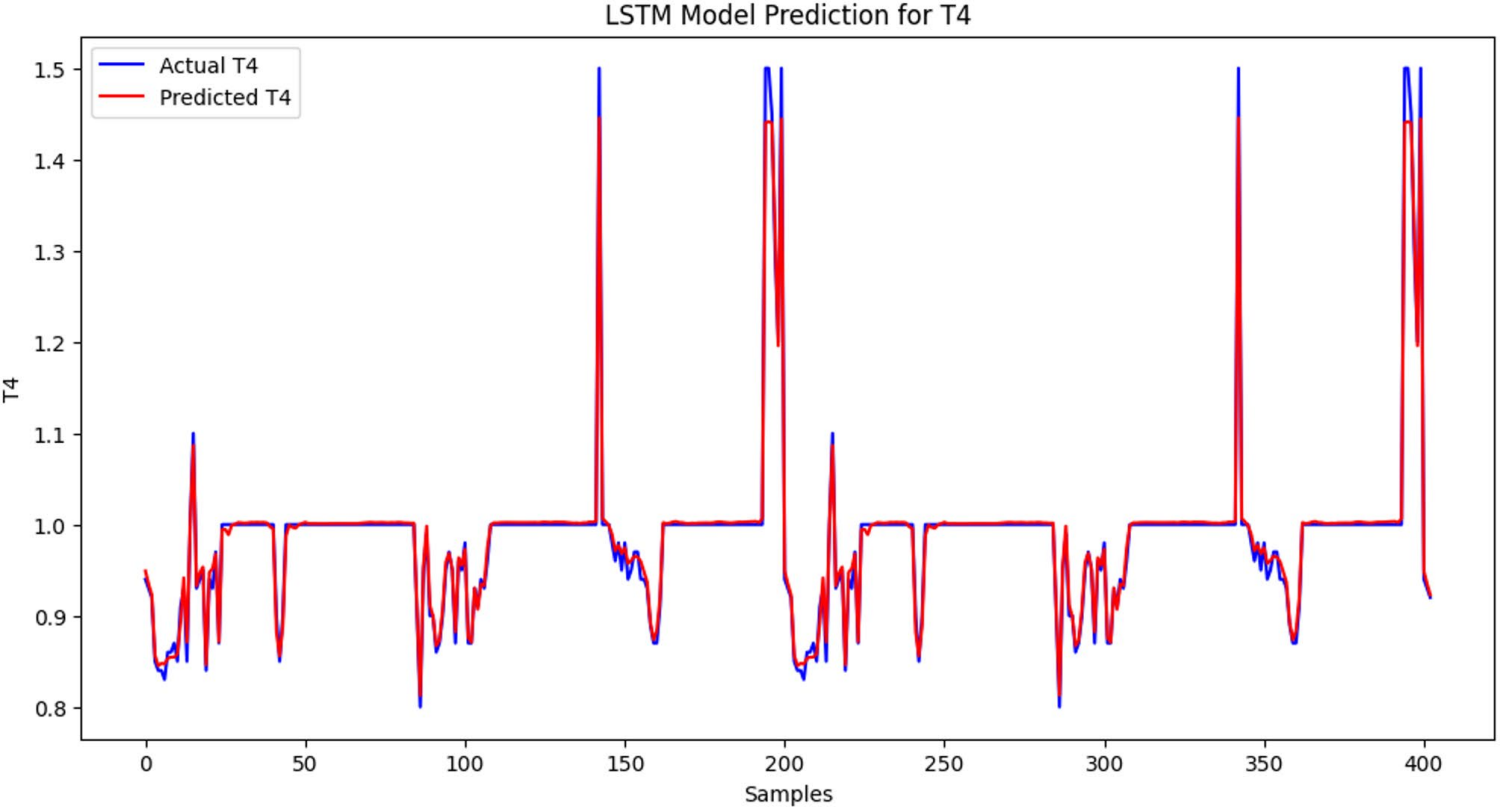
LSTM model prediction for T4 levels. The comparison of the actual (blue line) and LSTM-predicted (red line) T4 hormone levels of subclinical hypothyroid patients indicated that its performance was good (MSE = 0.0113). As depicted by the visualization, the model can capture both long-term changes and short-term changes in the level of T4

**Fig. 4:**
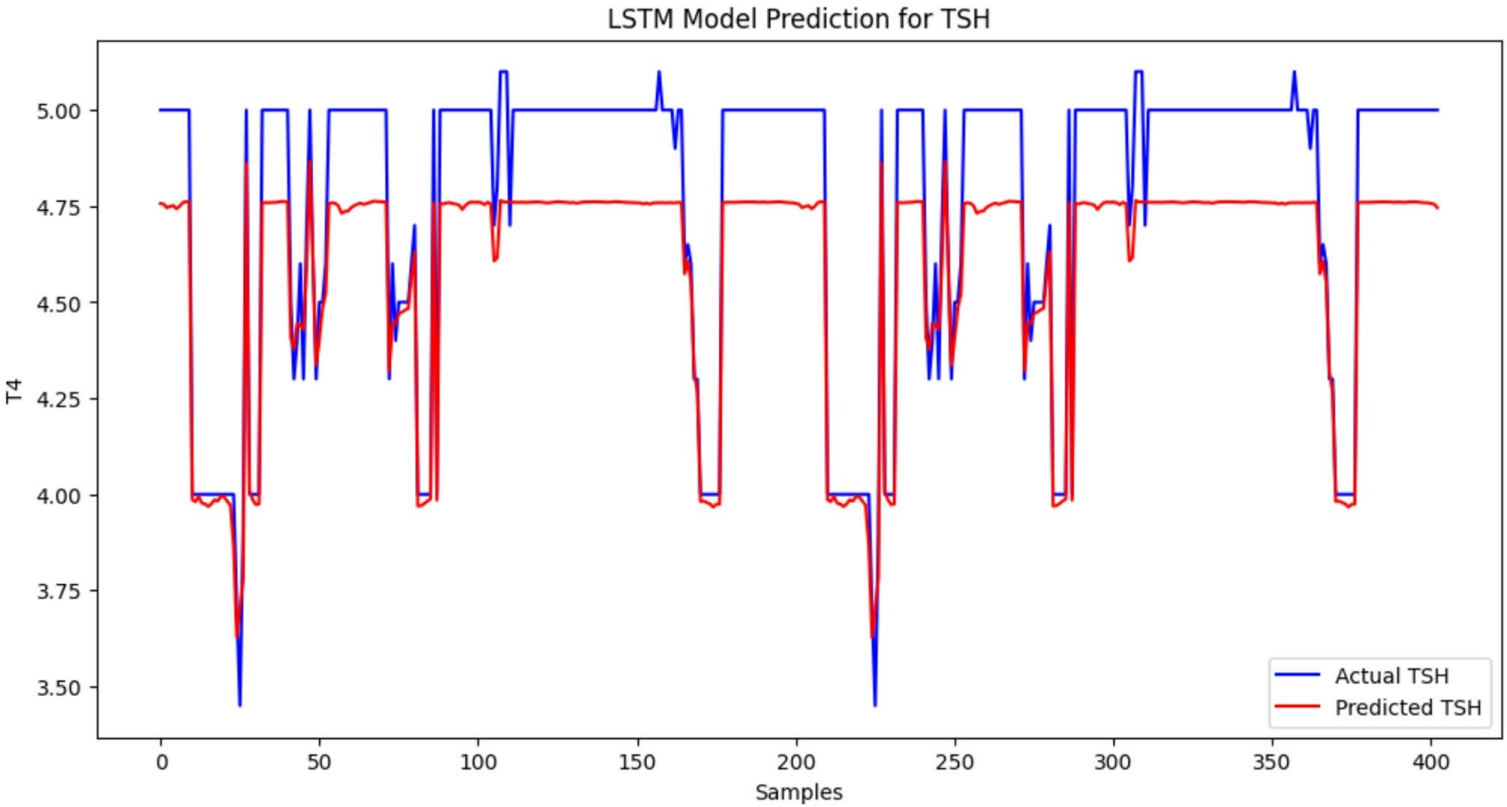
Results of the TSH level model. The last model yields a moderate prediction (MSE = 0.0426) because of the complexity of the dynamics of the TSH. The x-axis indicates a sequential sequence of data samples (not actual time), and the y-axis indicates the TSH concentration expressed in mU/L. A small sample of 400 is shown to present significant trends and patterns within the data to provide clarity. Among the notable characteristics are the specificities of the model to follow the general trends in TSH levels yet show some deviations in response to a quick change

The LSTM model performs well because the predicted values of the TyG index are near the actual values in most of the range. There are minor inconsistencies causing slight inaccuracies in the observed values compared with the expected values. The LSTM model demonstrated high predictive performance for TyG index levels, as the mean squared error (MSE) was low: 0.00034, which is an indicator of a low level of deviation between the predicted and actual values. The changes might also be explained by the fluctuations and differences in the data and shortcomings of the model in the ability to accommodate sudden spikes in the TyG index (Figure 1). The correlation between the predicted value and the actual value fits well, and the red line becomes closer to the blue line within the samples. The LSTM model is also very precise in predicting the T3 levels since the value of the MSE measure is quite low, indicating that the difference between the actual and approximated numbers is not large. The challenge with modelling this is that some abrupt changes in the scales, such as those of the shaking areas, were impossible to model, although the output of the data project was relatively contending compared with the actual quantity of T3. However, the model sensitivity, in general, in representing the concentration level of T3, indicates that it may be used in its capacity to reflect the degree of unstable thyroid hormones in patients with SCH (Figure 2). There has been proximity between the two lines, and the predictions have been quite on par with the actual values. MSE = 0.0113 is evidence that the LSTM model can predict FT4 levels quite accurately. The model can be useful in estimating most of the variants of FT4 and does not extend exactly to the fast-varying part, particularly in the spiky shoulders. Nevertheless, the overall findings of this model indicate that identifying patients with thyroid-related medical conditions might be beneficial with respect to the assessment of the level of FT4 (Figure 3). The blue trend represents the observed values of the TSH concentrations in the data, whereas the red trend depicts the predicted values of the TSH concentrations in the data modelled via the LSTM. The results are anticipated based on the general dynamics of the real data; however, there are some inconsistent figures in areas with drastic fluctuations. The findings provided by the LSTM model show that the prediction is average since the MSE is equal to 0.0426. This model functions well in reproducing the overall trend shifts in the TSH concentration but cannot be applied to reproducing instances where an abrupt decrease or increase in the TSH concentration occurs. Compared with previous histories of other hormones (T3, FT4), a relatively large-scale MSE suggests that further tuning is needed to refine the model to predict TSH in patients with thyroid-related disorders (Figure 4).

## Discussion

The correlation between the TyG index and thyroid function in patients with SCH is gaining increasing attention in metabolic and endocrine research. Subclinical hypothyroidism (SCH) with elevated TSH but normal FT4 concentration is frequently accompanied with defects in metabolic parameters, such as dyslipidaemia and IR, which are essential elements of the metabolic syndrome [23].

This study findings highlight clear sex- and age-specific differences. Females exhibited higher FPG, cholesterol, HDL, and TSH levels, while males demonstrated higher T3, liver enzymes, and hematological counts. Hormonal influences can explain these differences: estrogen enhances lipid metabolism and increases thyroid-binding proteins [24], whereas testosterone stimulates erythropoiesis and increases basal metabolic activity [25]. Age-related variations were also evident, with middle-aged individuals showing higher FPG and HbA1c, and triglyceride levels rising with advancing age. These results align with prior reports linking thyroid–metabolic interactions to sex and age [24, 26].

The triglyceride-glucose index (TyG) obtained from FPG and TG is an established surrogate marker for IR [27]. In this study, TyG was found to be significantly negatively associated with T3 levels and significantly positively associated with HbA1c, LDL and total cholesterol levels. These results indicate that SCH plays a greater role in lipid disturbance than glycemic imbalance, which again, is in agreement with reports that hypothyroidism increases TG by decreased lipoprotein lipase activity [28, 29]. Conversely, hyperthyroidism reduces TG by accelerating lipid clearance [28, 29].

Moreover, the findings of this study are consistent with other studies that demonstrated TyG is correlated with thyroid function even in euthyroid adults, in whom low-normal thyroid function is a marker of increased metabolic risk [30]. Data from the Korean National Health and Nutrition Examination Survey confirmed that TSH and FT4 predict TyG levels [30, 31]. However, other studies found no significant association between thyroid dysfunction and glucose control, suggesting that lipid metabolism may be the dominant driver of TyG elevations in SCH [8, 26, 32].

The metabolic abnormalities observed—elevated TG, LDL, and HbA1c—demonstrate that SCH is not a benign condition. Even in its subclinical form, hypothyroidism has been associated with insulin resistance and impaired glucose utilization [26, 33]. This highlights the TyG index as a simple, non-invasive tool for early identification of cardiometabolic risk in SCH. Incorporating TyG into routine screening may help detect high-risk patients earlier, enabling interventions such as lifestyle changes or levothyroxine therapy, which has been shown to improve lipid profiles in some cases [34].

In addition to TyG, our study demonstrated associations between thyroid function and hematological as well as hepatic parameters. Elevated liver enzymes in SCH patients may reflect non-alcoholic fatty liver disease, which is commonly linked to insulin resistance and dyslipidemia [35]. This supports previous evidence that thyroid hormones significantly influence lipid metabolism and liver activity [36]. Furthermore, BMI, body composition, and obesity are closely intertwined with thyroid function, reinforcing the role of SCH as a contributor to metabolic syndrome [37, 38].

### Strengths and limitations

The strengths of this study include its large sample size and the use of both regression analysis and LSTM, which confirmed the associations with high accuracy. However, limitations must be acknowledged: the cross-sectional design precludes causality, convenience sampling and gender imbalance may limit generalizability, and reliance on self-reported histories risks including undiagnosed metabolic conditions [23]. The study relied on self-reported medical history and clinical records as exclusion criteria, which may have led to the inclusion of participants with undiagnosed diabetes or dyslipidemia, potentially influencing the results.

## Conclusion

This study demonstrated that the TyG index is significantly associated with thyroid function parameters in subclinical hypothyroid patients, showing a negative relationship with T3 and positive associations with HbA1c and lipid markers. These findings indicate that the TyG index reflects thyroid-related metabolic alterations and may serve as a practical biomarker for assessing thyroid dysfunction risk. Clinically, its application could support earlier identification of patients at risk of progression and guide timely management. Further longitudinal studies are needed to validate these associations and establish population-specific reference values. By highlighting the role of the TyG index as a simple and cost-effective marker for the early detection of metabolic derangements in individuals with subclinical hypothyroidism, this study contributes to broader efforts to improve health equity, prevent chronic disease progression, and promote sustainable well-being in diverse populations.

## Supplementary Information

Supplementary Material 1.

## Data Availability

No datasets were generated or analysed during the current study.

## Abbreviations

ALP: Alkaline phosphatase
ALT: Alanine aminotransferase
AST: Aspartate aminotransferase
ATA: American Thyroid Association
BMI: Body mass index
CBC: Complete blood count
CVD: Cardiovascular disease
ECLIA: Electrochemiluminescence immunoassay
ETA: European Thyroid Association
FPG: Fasting plasma glucose
FT4: Free thyroxine
GGT: Gamma-glutamyl transferase
HbA1c: Hemoglobin A1c
HDL: High-density lipoprotein
HEC: Hyperinsulinaemic-euglycaemic clamp
HOMA-IR: Homeostatic model assessment of insulin resistance
IR: Insulin resistance
KTH: Khyber Teaching Hospital
LDH: Low-density lipoprotein cholesterol
LSTM: Long short-term memory
METS-IR: Metabolic score of insulin resistance
MSE: Mean squared error
NHANES: National Health and Nutrition Examination Survey
NICE: National Institute for Health and Care Excellence
PCOS: Polycystic ovary syndrome
QUICKI: Quantitative insulin sensitivity check index
SCH: Subclinical hypothyroidism
T2DM: Type 2 diabetes mellitus
TC: Total cholesterol
TG: Triglyceride
TH: Thyroid hormone
T3: Triiodothyronine
TSH: Thyroid-stimulating hormone
TyG index: Triglyceride–glucose index
VIF: Variance inflation factor
WBC: White blood cells

## References

[1] Li M, Chi X, Wang Y, Setrerrahmane S, Xie W, Xu H. Trends in insulin resistance: insights into mechanisms and therapeutic strategy. Signal Transduct Target Therapy. 2022;7(1):216. 10.1038/s41392-022-01073-0 PMC9259665 35794109

[2] Park SY, Gautier JF, Chon S. Assessment of insulin secretion and insulin resistance in human. Diabetes Metab J. 2021;45(5):641–54. 34610719 10.4093/dmj.2021.0220 PMC8497920

[3] Fasipe OJ, Ayoade OG, Enikuomehin AC, Falade CO. Evaluating antiretroviral therapy–induced insulin resistance syndrome using the homeostasis model assessment method: an important global Clarion call for concern among people living with HIV-disease. RPS Pharm Pharmacol Rep. 2024;3(3):rqae019.

[4] Bazyar H, Zare Javid A, Masoudi MR, Haidari F, Heidari Z, Hajializadeh S, Aghamohammadi V, Vajdi M. Assessing the predictive value of insulin resistance indices for metabolic syndrome risk in type 2 diabetes mellitus patients. Sci Rep. 2024;14(1):8917. 38632455 10.1038/s41598-024-59659-3 PMC11024148

[5] Cho YK, Han KD, Kim HS, Jung CH, Park JY, Lee WJ. Triglyceride-Glucose index is a useful marker for predicting future cardiovascular disease and mortality in young Korean adults: A nationwide Population-Based cohort study. J Lipid Atheroscler. 2022;11(2):178–86. 35656153 10.12997/jla.2022.11.2.178 PMC9133778

[6] Li HF, Miao X, Li Y. The triglyceride glucose (TyG) index as a sensible marker for identifying insulin resistance and predicting diabetic kidney disease. Med Sci Monit. 2023;29:e939482. 37421131 10.12659/MSM.939482 PMC10337482

[7] Mullur R, Liu YY, Brent GA. Thyroid hormone regulation of metabolism. Physiol Rev. 2014;94(2):355–82. 24692351 10.1152/physrev.00030.2013 PMC4044302

[8] Eom YS, Wilson JR, Bernet VJ. Links between thyroid disorders and glucose homeostasis. Diabetes Metab J. 2022;46(2):239–56. 35385635 10.4093/dmj.2022.0013 PMC8987680

[9] Biondi B, Kahaly GJ, Robertson RP. Thyroid dysfunction and diabetes mellitus: two closely associated disorders. Endocr Rev. 2019;40(3):789–824. 30649221 10.1210/er.2018-00163 PMC6507635

[10] Grice A. Subclinical hypothyroidism. InnovAiT. 2019;12(3):131–5.

[11] Pearce SH, Brabant G, Duntas LH, Monzani F, Peeters RP, Razvi S, Wemeau J-L. 2013 ETA guideline: management of subclinical hypothyroidism. Eur Thyroid J. 2013;2(4):215–28. 24783053 10.1159/000356507 PMC3923601

[12] Garber JR, Cobin RH, Gharib H, Hennessey JV, Klein I, Mechanick JI, Pessah-Pollack R, Singer PA, Woeber KA. Clinical practice guidelines for hypothyroidism in adults: cosponsored by the American association of clinical endocrinologists and the American thyroid association. Endocr Pract. 2012;18(6):988–1028. 23246686 10.4158/EP12280.GL

[13] Ma CG, Shim YS. Association of thyroid-Stimulating hormone and thyroid hormones with cardiometabolic risk factors in euthyroid children and adolescents aged 10–18 years: A Population-Based study. Sci Rep. 2019;9(1):15476. 31664103 10.1038/s41598-019-51963-7 PMC6820776

[14] Shin JA, Mo EY, Kim ES, Moon SD, Han JH. Association between lower normal free thyroxine concentrations and obesity phenotype in healthy euthyroid subjects. Int J Endocrinol. 2014;2014:104318. 24872812 10.1155/2014/104318 PMC4024385

[15] Canaris GJ, Manowitz NR, Mayor G, Ridgway EC. The Colorado thyroid disease prevalence study. Arch Intern Med. 2000;160(4):526–34. 10695693 10.1001/archinte.160.4.526

[16] Hollowell JG, Staehling NW, Flanders WD, Hannon WH, Gunter EW, Spencer CA, Braverman LE, Serum TSH. T4, and thyroid antibodies in the united States population (1988 to 1994): National health and nutrition examination survey (NHANES III). J Clin Endocrinol Metabolism. 2002;87(2):489–99. 10.1210/jcem.87.2.8182 11836274

[17] Emerson CH. Diagnosis and treatment of hypothyroidism: rules, longstanding exceptions, and the emerging entity of thyroid hormone receptor alpha resistance. Thyroid : official journal of the American Thyroid Association. 2012;22(12):1197–9. 10.1089/thy.2012.2212.ed. 10.1089/thy.2012.2212.ed 23210565

[18] Biondi B, Cooper DS. The clinical significance of subclinical thyroid dysfunction. Endocr Rev. 2008;29(1):76–131. 17991805 10.1210/er.2006-0043

[19] He H, Pan L, Cui Z, Sun J, Yu C, Cao Y, Wang Y, Shan G. Smoking prevalence, patterns, and cessation among adults in Hebei province, central china: implications from China National health survey (CNHS). Front Public Health. 2020;8:177. 32596196 10.3389/fpubh.2020.00177 PMC7300263

[20] Zierle-Ghosh A, Jan A. Physiology, Body Mass Index. In: StatPearls. edn. Treasure Island (FL): StatPearls Publishing Copyright © 2025, StatPearls Publishing LLC.; 2025. 30571077

[21] Liu C, Liang D. The association between the triglyceride-glucose index and the risk of cardiovascular disease in US population aged ≤ 65 years with prediabetes or diabetes: a population-based study. Cardiovasc Diabetol. 2024;23(1):168. 38741118 10.1186/s12933-024-02261-8 PMC11092030

[22] Simental-Mendía LE, Rodríguez-Morán M, Guerrero-Romero F. The product of fasting glucose and triglycerides as surrogate for identifying insulin resistance in apparently healthy subjects. Metab Syndr Relat Disord. 2008;6(4):299–304. 19067533 10.1089/met.2008.0034

[23] Alsulami SS, Baig M, Albeladi AH, Alyoubi SB, Alsubaie SA, Albeladi SA, Ghamri KA, Alraiqi AMS, Alyoubi SM, Almutairi WA. Correlation between subclinical hypothyroidism and metabolic syndrome: A retrospective study. Saudi J Med Med Sci. 2023;11(3):250–6. 37533656 10.4103/sjmms.sjmms_225_22 PMC10393097

[24] Lauretta R, Sansone M, Sansone A, Romanelli F, Appetecchia M. Gender in endocrine diseases: role of sex gonadal hormones. Int J Endocrinol. 2018;2018:4847376. 30420884 10.1155/2018/4847376 PMC6215564

[25] Li X, Meng Z, Tan J, Liu M, Jia Q, Zhang G, He Y, Zhang Q, Liu L, Song K, et al. Gender impact on the correlation between thyroid function and serum lipids in patients with differentiated thyroid cancer. Exp Ther Med. 2016;12(5):2873–80. 27882089 10.3892/etm.2016.3701 PMC5103717

[26] Wang C-Y, Chang T-C, Chen M-F. Associations between subclinical thyroid disease and metabolic syndrome. Endocr J. 2012;59(10):911–7. 22785370 10.1507/endocrj.ej12-0076

[27] Lee DY, Lee ES, Kim JH, Park SE, Park CY, Oh KW, Park SW, Rhee EJ, Lee WY. Predictive value of triglyceride glucose index for the risk of incident diabetes: A 4-Year retrospective longitudinal study. PLoS ONE. 2016;11(9):e0163465. 27682598 10.1371/journal.pone.0163465 PMC5040250

[28] Hashim AM, Humadi AT. Hypothyroidism, hyperthyroidism and its relationship with lipid profile in thyroid dysfunction patients. J Univ Babylon Pure Appl Sci. 2023;31(1):122–33.

[29] Shin KA, Kim EJ. Association between thyroid hormone and components of metabolic syndrome in euthyroid Korean adults: A population-based study. Med (Baltim). 2021;100(51):e28409. 10.1097/MD.0000000000028409 PMC8701466 34941185

[30] Choi W, Park JY, Hong AR, Yoon JH, Kim HK, Kang HC. Association between triglyceride-glucose index and thyroid function in euthyroid adults: the Korea National health and nutritional examination survey 2015. PLoS ONE. 2021;16(7):e0254630. 34264998 10.1371/journal.pone.0254630 PMC8281995

[31] Choi YM, Kim MK, Kwak MK, Kim D, Hong EG. Association between thyroid hormones and insulin resistance indices based on the Korean National health and nutrition examination survey. Sci Rep. 2021;11(1):21738. 34741077 10.1038/s41598-021-01101-z PMC8571382

[32] Sakyi SA, Ameyaw B, Laing EF, Anthony R, Ephraim RKD, Effah A, Kwayie AA, Senu E, Anto EO, Acheampong E. Thyroid dysfunction and glycaemic control among type 2 diabetes mellitus patients in ghana: A comparative cross-sectional study. Endocrinol Diabetes Metabolism. 2023;6(6):e447. 10.1002/edm2.447 PMC10638622 37621219

[33] Gronich N, Deftereos SN, Lavi I, Persidis AS, Abernethy DR, Rennert G. Hypothyroidism is a risk factor for New-Onset diabetes: A cohort study. Diabetes Care. 2015;38(9):1657–64. 26070591 10.2337/dc14-2515

[34] Razvi S, Weaver JU, Butler TJ, Pearce SHS. Levothyroxine treatment of subclinical hypothyroidism, fatal and nonfatal cardiovascular events, and mortality. Arch Intern Med. 2012;172(10):811–7. 22529180 10.1001/archinternmed.2012.1159

[35] Xie Y, Wang Z, Chen Z. Analysis of Subclinical Thyroid Dysfunction and Metabolic Abnormality in 28568 Healthy People. *Int J Endocrinol* 2023, 2023:5216945. 10.1155/2023/5216945 PMC10593554 37876378

[36] Wang L, Chen T, Yu J, Yuan H, Deng X, Zhao Z. Clinical associations of thyroid hormone levels with the risk of atherosclerosis in euthyroid type 2 diabetic patients in central China. Int J Endocrinol. 2020;2020:2172781. 32714391 10.1155/2020/2172781 PMC7354656

[37] Yin J, Wang C, Shao Q, Qu D, Song Z, Shan P, Zhang T, Xu J, Liang Q, Zhang S, et al. Relationship between the prevalence of thyroid nodules and metabolic syndrome in the Iodine-Adequate area of hangzhou, china: A Cross-Sectional and cohort study. Int J Endocrinol. 2014;2014:675796. 25197276 10.1155/2014/675796 PMC4150509

[38] Han C, He X, Xia X, Li Y, Shi X, Shan Z, Teng W. Subclinical hypothyroidism and type 2 diabetes: A systematic review and Meta-Analysis. PLoS ONE. 2015;10(8):e0135233. 26270348 10.1371/journal.pone.0135233 PMC4535849

