# Supplementary material for: Exploring the associations of the triglyceride‒glucose index with thyroid function in subclinical hypothyroid patients: a cross-sectional study
Source: Lipids Health Dis. 2025 Oct 21;24:337. doi: 10.1186/s12944-025-02740-1 (PMC12539201; doi:10.1186/s12944-025-02740-1)
Supplement: Supplementary file 1 — Supplementary Material 1. [file 12944_2025_2740_MOESM1_ESM.pdf]

Exploring the associations of the triglyceride–...

By: Fawad Inayat

As of: Sep 5, 2025 9:18:08 AM
6,608 words - 98 matches - 80 sources

Similarity Index

14%

Mode: Similarity Report

paper text:

Exploring the

associations of the triglyceride–glucose index 77

with thyroid function in subclinical hypothyroid patients: **A cross-sectional study** 49

Abstract: Background: Subclinical hypothyroidism (SCH) often occurs concomitantly with the emergence of the metabolic disorder

insulin resistance (IR). This study aimed to determine **the relationship between** the Triglyceride Glucose (**TyG**) index **and thyroid function in** 30

patients with subclinical hypothyroidism (SCH), to identify metabolic predictors of thyroid dysfunction.

Methods: This cross-sectional study used convenience sampling 57

, and data were collected after written informed consent. This

study was conducted at tertiary care hospitals in Peshawar, Pakistan, **and** 59

included 2024 subclinical hypothyroid patients with an age>19 years. Individuals with any thyroid condition, diabetes, cardiovascular disorders or chronic liver conditions were excluded. Regression, ANOVA, and long short-term memory (LSTM)

models were used to predict **the** TyG index, **TSH** , T3, **and FT4** 27

levels.

All analyses were performed **using R version 4.3.0** 11

and Python. The result was considered statistically significant with

P < 0.05. Results: The male-to-female ratio was 1:2 37

, and the highest group included 41 50-year-olds (40.3%). Regression analysis revealed

an inverse association **between the TyG index and** 68

T3 level (

β = -0 .313, **P < 0** .0001) **and a positive association with** HbA1c (**β= 0** .198; **P < 0** 33

.0001), indicating

a relationship between a higher **TyG index and** 70

IR and poor glycemic control. The values of HDL were negatively correlated with the TyG index (β = -0.221, P < 0.0001); conversely, LDL

was positively correlated to TyG (β = **0** .234, **P < 0** 1

.0001). The LSTM model presented high predictive accuracy with small mean squared errors, 0.00034 for the TyG index, 0.0015 for T3, and 0.0113 for T4. Conclusion: The findings of this study

**demonstrated that the TyG index can be an** effective and **important** parameter **of metabolic**	6

health and a predictor of thyroid function in subclinical hypothyroid 2 patients. These findings underscore the importance of early identification of metabolic risk factors for thyroid dysfunction, which can contribute to improved health outcomes and reduce the long-term burden of endocrine and cardiovascular diseases at the population level. Moreover, the results of the current research cannot be generalized, as it is a cross-sectional study. Keywords: Triglycerides; Glucose; Insulin resistance; Thyroid gland/physiopathology; Hypothyroidism; Subclinical Introduction: Insulin resistance (IR): a condition where resistance to respond to insulin exists. However, a high BMI is also an unfavorable predisposing risk factor for multiple metabolic complications

**such as type 2 diabetes mellitus (T2DM), cardiovascular** disease (**CVD), polycystic ovary syndrome (PCOS), and**	32

dislipidemia [1].

**The hyper-insulinaemic- euglycaemic clamp** (HEC) **is the** gold- **standard** of measuring **IR**	50

. The complexity, expense and time of using this method have restricted its use in the clinic and research setting [2, 3]. In addition, pseudo-indices

**such as the homeostatic model assessment of insulin resistance (HOMA-IR), metabolic score** of **insulin resistance (METS-IR**), quantitative insulin sensitivity **index** (QUICKI) **and**	3

Matsuda index are also simulated [4].

**Among these indices, the** triglyceride–glucose (**TyG) index**	80

, employs only two parameters, i.e.,

**fasting plasma glucose (FPG) and** triglyceride (**TG**) concentrations and **has**	67

gained increased attention because of its simplicity and applicability as a potential predictor of IR and other metabolic disorders [5, 6]. The inconsistency in many metabolic processes is associated with the thyroid hormone (THs) and glucose metabolism [7]. Hyperthyroidism is caused by excess synthesis and secretion of THs and is characterised by irregular insulin signalling [8]. Moreover, hypothyroidism is directly linked with metabolic disorders, such as IR [8, 9]. SCH is defined by the following set of biochemical features: increased serum levels of

thyroid-stimulating hormone (TSH) and normal levels **of free thyroxine (FT4**). Generally, **subclinical hypothyroidism is defined as TSH** 31

> 4.

0 mIU/L, depending on which country **the** 75

patient is screened in and which is

the National Institute for Health and Care Excellence (NICE 55

) [10], Although similar thresholds are used

by the European Thyroid Association (ETA) and American Thyroid Association (ATA), in 20

general,

TSH levels between **4** .0- **10** .0 **mIU/L** 76

are commonly regarded as mild SCH [11, 12]. Persistent hyperthyroidism is confirmed over time, and symptomatic patients or patients with TSH >10 mIU/L are treated in most cases. However,

studies investigating the correlation **between thyroid function and** IR **have** 27

shown contradictory results, and there are

few studies that **have investigated the** associations **between TyG index and thyroid** function **in** 40

patients with SCH [13, 14].

The present study was undertaken **to test the hypothesis that the** 19

TyG index is significantly **correlated with** thyroid function **in patients with** 26

SCH. While previous studies have investigated insulin resistance and metabolic disturbances in thyroid disorder patients, there is limited evidence about the presence of TyG index as a surrogate index in this population. This study adds to the existing knowledge by being among

the first to systematically evaluate **the** relationship **between the TyG index and** TH **in** SCH **patients** 42

and by employing both regression models and LSTM, thereby highlighting the potential

utility of the TyG index as a novel indicator **of metabolic** 54

risk and thyroid-related complications. Materials

and methods Study settings **and design This** observational **cross-sectional study was conducted in** 38

three

tertiary care hospitals namely **Hayatabad Medical Complex (HMC), Khyber Teaching Hospital** 24

(KTH), and Lady Reading Hospital (LRH) in urban area of Peshawar

,

Pakistan from January 2022 to August 2022 . The sampling technique was 62

easily determined and conceptualised to introduce the participants to the study information, orally. Written informed consent was received to take part

in the study. Inclusion criteria ? Adults aged 18–65 years 45

. ? SCH participants were indicated as having high TSH and normal FT4. ? Individuals who have not been previously diagnosed with DM, CVD, cerebrovascular diseases, malignant conditions or any chronic liver conditions. Exclusion criteria ? Women who are pregnant ? Patients with confirmed thyroid disorders other than SCH. ? Patients with or without lipid-lowering therapy. Sample size The final

sample size (2024) was calculated using the expected prevalence of SCH in 61

the target population, as it was reported to be 4–10% among a general adult population [15, 16]. This estimation ensured the sufficiency of statistical power (100%) to achieve clinically significant estimations of the primary outcomes, which included the parameters of thyroid function and metabolic indicators, in this case,

at a significance level of P = 0.05 21

. There is reliance on prior effect sizes in the discussion of the power observed within comparable populations, and this reliance extends to diagnostic criteria, as noted in its inclusion criteria [17, 18]. Demographic, lifestyle, and anthropometric parameters

Data including demographic information (age, sex, height, weight and 9

BMI) were collected [19].

BMI was computed **as the ratio of** kg **to height squared (m2** 19

) [20]. Measurement of laboratory indices Sampling of the blood was done in the morning and a minimum of 8 hours fasting was observed prior to the blood being collected. The gel tube was filled with five ml of blood into a 22-gauge needle/5 ml syringe into which blood was collected. An FT4 and TSH serum was analysed using a C501 analyser (Roche Diagnostics). These analyses were conducted through the concept of electrochemiluminescence immunoassays (ECLIAs). Blood FPG and HbA1c were tested. The HgA1c was determined using High-Performance liquid Chromatography (HPLC) method. A Cobas 6000 Roche diagnostics analyser was used to validate it.

TyG index was computed using the natural logarithm of **fasting triglycerides** 26

and multiplying it with the natural logarithm of fasting glucose and dividing the two logarithms by 2. In

TyG = ln (fasting triglyceride (**mg/dl)fasting glucose (mg/dl)/2 [21** 5

, 22].

The total serum cholesterol , TG, **high- density lipoprotein (HDL), low-density lipoprotein (LDL), and** 29
FPG **levels** of the participants **were**

also determined. Statistical analysis The mean difference among groups of continuous variables was determined using ANOVA,

and the chi-square test was used **for categorical variables. Multivariate linear regression was** 35
utilized **to**

study the continuous outcomes and was adjusted for confounding variables and quantify the associations. The problem of multicollinearity of predictors (TyG index, sex, age, BMI) has also been evaluated as a variance inflation factor (VIF). The VIFs of all the variables were considerably lower (<2) than the recommended cut-off of 5, suggesting a lack of multicollinearity. The regression of the TyG index with thyroid function was performed via a multivariable linear regression model, and all the regulative-related requirements to regress, including normality, were assumed to be met. In the present

study, an LSTM model that uses input one-time series data (sequence one), an LSTM layer with 64 cell units, a 0.2 dropout ratio, and one-unit output was used. This was accomplished through progressive, progressive and emergent theoretical learning models, that is, through Adam optimization (

learning rate = 0.001) and **loss** , the **mean squared error** (MSE). **The** 53

data were divided into training and test sets 4

at proportions of 80--20, and 5-fold cross-validation was performed to ensure that the model used was not overtrained.

The analyses were performed via **R and** 1

Python, with $P < 0.05$ regarded as the significance level.

Ethical considerations The research was approved by the Research **Ethical Committee** 47

(REC) at Iqra National University, Peshawar, Pakistan (No. INU/AHS/57-22).

Written informed consent was obtained from all participants prior to data collection. Results Baseline 16
characteristics **of**

the participants The demographic baseline description of the study population provided some critical aspects. The study comprised 2024 participants, and a larger portion of them are female (66.9%). The age group 41-50 years is the prominent one (40.3%), followed by the 51-60 years (27.5%). The mean BMI (25.09±1.12) of the participants is indicative of overweight. Thyroid function tests revealed that the mean level of TSH was slightly greater (4.77±0.38). The lipid profile indicated elevated cholesterol and LDL compared with HDL, which is likely a risk factor for cardiovascular issues. The TyG index mean value in the study population is 4.80, which exceeds the standard cut-off point (4.67) identified by Simental-Mendia et al. (2008) [23]. The liver function tests (

alanine aminotransferase (ALT), alkaline phosphatase (ALP), aspartate aminotransferase (AST), and 25

gamma-glutamyl transferase (GGT)) and Complete Blood Count (CBC

) were normal [

Table 1]. Table 1: Baseline characteristics of the study participants Characteristics Values 39

Sex (%) Male 670 (33.1%) Female 1354 (66.9%) Age Group (Years) 21-30 Years 128 (6.3%) 31-40 Years 376 (18.6%) 41-50 Years 815 (40.3%) 51-60 Years 556 (27.5%) 61-70 Years 149 (7.4%) Height (Feet) 5.44 ± 0.244 Weight (Pounds) 148.63 ± 10.74 Body Mass Index (BMI) 25.09 ± 1.12 TG 163.91 ± 17.33 TSH (Milliunit/Liter) 4.77 ± 0.38 FT4 (ng/dl) 0.99 ± 0.097 T3 (pg/ml) 1.67 ± 0.44 Cholesterol (mg/dl) 256.38 ± 49.18 LDL (mg/dl) 130.093 ± 27.54 HDL (mg/dl) 42.82 ± 13.96 HbA1c 6.59 ± 0.50 FPG (mg/dl) 90.80 ± 9.69 TyG Index 4.80 ± 0.069 ALT (U/L) 39.72 ± 27.71 ALP (U/L) 126.63 ± 60.76 AST (U/L) 46.40 ± 18.73 GGT (U/L) 58.35 ± 17.05 RBC (million cells/μL) 4.27 ± 0.85 WBC (thousand cells/μL) 4.44 ± 0.73 197 Gender Differences in the Metabolic and Thyroid Profiles of Subclinical Hypothyroid Patients This study compared demographic, lipid, thyroid, liver, and hematological parameters. The average weights and heights of the males (161.09±9.39 pounds and 5.71±0.20 feet, respectively) were greater than females (142.47±3.76 pounds and 5.31 ± 0.13 feet,

respectively; P<0.001). The female exhibited higher **FPG** 1

(92.21±10.91

mg/dl, P<0.001), cholesterol 1

(262.66 ± 44.56

mg/dl; P< 0.001), HDL 78

(44.84±14.25

mg/dl; P < 0.001), LDL 1

(133.54±25.40

mg/dl; P < 0.001), and	1

TSH (4.85±0.28 milliunits/L; P<0.001) compared to males. All these differences in metabolic and thyroid profiles are due to hormones: estrogens in women stimulate lipid metabolic activities and inhibit thyroid-binding proteins, while testosterone in men stimulates the metabolic rate and hepatic enzyme synthesis. However, the finding revealed that the males have higher concentration of T3 (2.05 ± 0.32 pg/ml; P<0.001), ALT (49.59 ± 31.34 U/L; P<0.001), GGT (65.07 ± 15.72 U/L; P<0.001), RBC counts (4.65±0.65 million

**cells/µl; P<0.001) and**	36

WBC counts (4.53±0.61 thousand

**cells/µl; P<0.01) than**	36

females. The TyG index did not differ significantly between sexes (

**P >0.05) (Table 2). Table 2: Gender**	-Based Comparison	**of**	Metabolic, Thyroid,	**and**	46

Haematological Parameters among Subclinical Hypothyroid Patients Parameter Male: Mean (SD) Female: Mean (SD) Levene's Statistic P value BMI (kg/m²) 24.83 (1.05) 25.23 (1.13) 3.43 0.064 Weight (pounds) 161.09 (9.39) 142.47 (3.76) 493.68 < 0.001* Height (feet) 5.71 (0.20) 5.31 (0.13) 277.16 < 0.001* FPG (mg/dl) 87.93 (5.53) 92.21 (10.91) 152.85 < 0.001* HbA1c (%) 6.59 (0.49) 6.59 (0.50) 1.26 0.262 TG (mg/dl) 159.57 (19.81) 166.05 (15.53) 56.34 < 0.001* Cholesterol (mg/dl) 243.70 (55.29) 262.66 (44.56) 171.24 <0 .001* TSH (milliunit/L) 4.61 (0.49) 4.85 (0.28) 198.52 <0 .001* T3 (pg/ml) 2.05 (0.32) 1.48 (0.37) 39.75 < 0.001*

**FT4 (ng/dl) 1.01 (0**	.14)	**0**	.98 (	**0**	.06) 86.1 <	**0.001**		48

* TyG Index (unitless) 4.77 (0.07) 4.81 (0.07) 1.83 0.176 HDL (mg/dl) 38.72 (12.41) 44.84 (14.25) 35.64 < 0.001* LDL (mg/dl) 123.12 (30.27) 133.54 (25.40) 38.69 < 0.001* ALT (U/L) 49.59 (31.34) 34.84 (24.30) 133.42 < 0.001* ALP (U/L) 113.19 (54.72) 133.28 (62.50) 86.35 < 0.001* GGT (U/L) 65.07 (15.72) 55.03 (16.71) 6.62 0.01* WBC (thousand cells/µL) 4.53 (0.61) 4.39 (0.78) 10.01 0.002* RBC (million cells/µL) 4.65 (0.65) 4.08 (0.87) 263.27 < 0.001* Note: *

A P value less than 0.05 was considered statistically significant	2

. 216 217 Age-related variations in the metabolic, thyroid, and haematological profiles of 218 subclinical hypothyroid patients 219 Age-wise, the analysis revealed significant changes in metabolic, thyroid, and haematological 220 data. BMI and weight decrease with increasing age, and younger patients (21--30 years) had the highest BMI (25.46 ± 1.11 kg/m2;

P<0.001) and weight (P<0.001) compared	52

with older patients (61--70 years). The height gain with age was slight, particularly beyond the ages of 51--60 years and 61--70 years, and highly variable (P<0.001). The FPG reached a maximum in groups 31--40 (95.05 ± 12.74

mg/dl; P<0.001) and decreased significantly in the older age group	1

. Similarly, the maximum HbA1c

was observed in the middle -aged group	1

(31--40 years), whereas the lowest level was found

in the younger and older groups (P<0.001). TG levels significantly increased	56

with age (168.49±21.93 mg/dl; P<0.001), suggesting that lipid metabolism becomes more complex with increasing age. The cholesterol levels, however, exhibited very different trends, with the youngest age group having the highest cholesterol levels and decreases in old age group (P<0.001), while the TSH remained the same among the age groups (P=0.568). However, the mean T3 and FT4 values are significantly different, whereas T3 was found to be higher at the youngest age, and FT4 was unstable with age (P< 0.001). Minor but notable variations were observed in the TyG index across the age group (P<0.001). The HDL level increases, and LDL decreases with an increase in age (P<0.001), which implies that there might be an age-related difference in the level of lipids. The changes in the levels of liver enzymes (ALT, ALP, GGT) and hematological parameters (WBC, RBC) also differed in terms of age (P<0.001) [Table 3]. 244 245 Table 3: Age-based differences in metabolic, thyroid, and hematological parameters among subclinical hypothyroid patients Parameter

21-30: Mean (SD) 31-40: Mean (SD) 41-50: Mean (SD) 51-60: Mean (SD) 61 -70: **Mean (SD**) F- 18
statistic **P value**

BMI (kg/m²) 25.46 (1.11) 25.12 (1.08) 25.19 (1.24) 25.00 (0.95) 24.61 (0.90) 13.29 < 0.001* Weight (pounds) 150.75 (12.32) 147.02 (10.36) 148.08 (9.67) 149.82 (11.12) 149.49 (13.37) 5.9 < 0.001* Height (feet) 5.44 (0.32) 5.40 (0.23) 5.43 (0.23) 5.47 (0.24) 5.48 (0.27) 6.39 < 0.001* Fasting Sugar Level (mg/dl) 91.51 (8.10) 95.05 (12.74) 89.80 (9.41) 89.95 (7.63) 88.02 (6.90) 25.7 < 0.001* HbA1c (%) 6.24 (0.33) 6.62 (0.49) 6.68 (0.50) 6.59 (0.49) 6.35 (0.43) 33.99 < 0.001* Triglyceride (mg/dl) 152.98 (18.83) 159.76 (14.09) 165.60 (16.65) 165.52 (17.12) 168.49 (21.93) 25 < 0.001* Cholesterol (mg/dl) 273.75 (30.76) 254.26 (44.37) 257.90 (48.50) 257.11 (52.58) 235.79 (56.92) 11.14 < 0.001* TSH (milliunit/L) 4.76 (0.43) 4.75 (0.39) 4.78 (0.38) 4.77 (0.37) 4.81 (0.40) 0.74 0.568 T3 (pg/ml) 1.87 (0.36) 1.55 (0.50) 1.69 (0.45) 1.67 (0.40) 1.69 (0.38) 14.41 < 0.001*

FT4 (ng/dl) 0 .97 (**0** .04) **0.99 (0** .10) **0** .97 (**0** 22

.05) 1.03 (0.14) 0.94 (0.05) 41.9 < 0.001* Triglyceride Glucose Index (unitless) 4.77 (0.06) 4.81 (0.07) 4.80 (0.07) 4.80 (0.07) 4.80 (0.08) 7.78 < 0.001* HDL (mg/dl) 43.59 (7.47) 39.52 (9.17) 40.58 (12.49) 44.83 (16.17) 55.19 (18.45) 46.57 < 0.001* LDL (mg/dl) 141.51 (8.96) 134.42 (23.32) 130.03 (27.39) 128.72 (30.87) 114.87 (28.60) 20.3 < 0.001* ALT (U/L) 45.70 (26.32) 35.52 (30.63) 45.74 (27.61) 34.45 (24.15) 31.96 (26.60) 22.09 < 0.001* ALP (U/L) 173.10 (55.44) 117.80 (60.93) 128.60 (55.01) 116.14 (60.47) 137.34 (73.86) 27.6 < 0.001* GGT (U/L) 69.48 (10.77) 55.01 (17.58) 58.76 (16.28) 57.06 (17.85) 59.79 (17.14) 19.09 < 0.001* WBC (thousand cells/µL) 4.41 (0.52) 4.91 (0.93) 4.25 (0.66) 4.37 (0.61) 4.59 (0.54) 62.23 < 0.001* RBC (million < 0.001* cells/µL) 4.33 (0.80) 4.66 (0.67) 4.13 (0.90) 4.21 (0.83) 4.19 (0.77) 28.35 Note:

A P value less than 0.05 was considered statistically significant. Correlation analysis 15

of metabolic, thyroid, and biochemical parameters in subclinical hypothyroid patients Spearman correlation showed that the associations of metabolite with thyroid and biochemical parameters were significant in SCH patients. The TyG was well

correlated with FPG (**r = 0** .635, **P < 0** .001) **and** moderately **correlated with** HbA1c (8
r = 0 .220, **P < 0** .001). **The** cholesterol value **was** significantly **correlated with** LDL (
r = 0 .856, **P<0** .001) **and** negatively with HDL (**r = -0** .544, **P<0**

.001) which reflects a desirable lipid pattern in the patient with subclinical hypothyroidism. The concentration of cholesterol

correlated positively with TSH (r = 0 .303, P<0.001 | 22

), suggesting that there is a reciprocal relation between cholesterol and TSH. TSH

positively correlated with HbA1c (**r = 0.286, P<0.001) and negatively correlated with** BMI (**r = -0** .354, **P<0.001** | 13

), which may be related to poor glycemia and low BMI in these patients. T3

was negatively correlated with cholesterol (**r = -0.189, P<0.001) and positively correlated with** ALT (**r = 0** .287, **P<0.001** | 23

), which suggested some complicated relationships between TH, lipoprotein metabolism and liver enzymes [

Table. 4]. 258 **Table 4** : Spearman **correlation analysis of** | 72

metabolic, thyroid, and biochemical parameters in subclinical hypothyroid patients Parameter Det ails T G HbA1 c FPG TyG index T3 FT4 TSH Chole sterol BMI Weight LDL HDL ALT ALP AST TG (mg/dl) P.C 1 0.058* * - 0.132* * 0.678* * - 0.168* * - 0.033* * 0.078* * 0.241** 0.071* * - 0.176* * 0.086* * -0.088** -0.033 -.053* - 0.076** Sig. 0.009 0.0001 0.0001 0.0001 0.0001 0.0001 0.0001 0.001 0.0001 0.0001 0.0001 0.138 0.018 0.001 HbA1c P.C 1 0.224* * 0.220* * -0.262 0.094* * 0.130* * 0.286** 0.284* * -0.015 0.270* * -0.223 - 0.123* * -0.301** - 0.165** Sig. 0.0001 0.0001 0.0001 0.0001 0.0001 0.0001 0.0001 .497 0.0001 0.0001 0.0001 0.0001 0.0001 FPG (mg/dl) P.C 1 0.635* * - 0.458* * -0.010 -0.002 0.146** 0.188* * - 0.109* * 0.244* * -0.103** -0.025 0.088** - 0.219** Sig. 0.0001 0.0001 .639 .932 0.0001 0.0001 0.0001 0.0001 0.0001 .269 0.0001 0.0001 TyG index P.C 1 .472** -.020 .063** .306** .187** -.226** .257** -.150** -.054* .020 -.219** Sig. 0.0001 0.372 0.005 0.0001 0.0001 0.0001 0.0001 0.0001 .016 .377 0.0001 T3 (pg/ml) P.C 1 0.159* * -0.003 -0.189** - 0.191* * 0.433* * - 0.266* * 0.002 0.179* * 0.092** 0.287** Sig. 0.0001 0.907 0.0001 0.0001 0.0001 0.0001 0.916 0.0001 0.0001 0.0001 FT4 (ng/ml) P.C 1 0.096* * -0.006 -0.035 -0.027 0.005 -0.026 0.016 -0.023 -0.002 Sig. 0.0001 0.796 0.111 0.220 0.822 0.247 0.464 0.311 0.945 TSH (milliunit/L) P.C 1 0.303** 0.060* * - 0.354* * 0.171* * -0.042 - 0.081* * 0.093** - 0.053** Sig. 0.0001 0.007 0.0001 0.0001 0.062 0.0001 0.0001 0.010 Cholesterol (mg/dl) P.C 1 0.136* * - 0.169* * 0.856* * -0.544** - 0.294* * 0.026 0.104** Sig. 0.0001 0.0001 0.0001 0.0001 0.0001 .283 0.0001 BMI P.C 1 - 0.141* * 0.079* * -0.163** 0.144* * -0.089** - 0.062** Sig. 0.0001 0.0001 0.0001 0.0001 0.0001 0.006 Weight (Pounds) P.C 1 - 0.058* * -0.108** 0.101* * -0.018 0.015 Sig. 0.0001 0.0001 0.0001 0.421 0.512 LDL (mg/dl) P.C 1 -0.566** - 0.391* * 0.077** 0.053* Sig. 0.0001 0.0001 0.0001 0.018

HDL (mg/dl) P.C 1 0.059* * 0.049* 0.047* Sig. 0.008 0.026 0.036 ALT (U/L) P.C 1 0.227** -0.050* Sig. 0.0001 0.023 ALP (U/L) P.C 1 0.490** Sig. 0.0001 **

A P value less than 0 .001 **was considered statistically significant** . PC; Pearson **Correlation** Sig; Significance Regression **analysis** 15

of

the TyG index, sex, age , and **BMI** on various metabolic **and** 69

thyroid parameters The regression model revealed that

the associations between the TyG index and sex, age, **BMI, and** 51

other metabolic and thyroid parameters in subclinical hypothyroid patients were strong. The major predictors of FT4 included female sex, whose levels were lower (β = -0.150, P<0.0001), and the TyG index; age and BMI were not notable predictors. T3 levels also showed a significant inverse

correlation with the TyG index (B = - **0** .313; **P<0** 60

.0001). Males have an insignificant

association with T3 (**β = -0** .501, **P<0** 74

.0001), with a negative correlation with BMI. TSH was a strong positive predictor since females had higher values (β = 0.314, P < 0.0001). The HbA1c

was positively associated with the TyG index (β 12

= 0.198, P < 0.0001),

indicating that a higher TyG index 79

would imply worsening glycemic control. The LDL (

β = 0 .234, **P < 0** .0001), **and** HDL (**β=-0** .221, **P<0** .0001) **were significantly associated with** 34

the TyG index. In case of cholesterol, **the TyG index** 71

is a strong positive predictor (β = 0.273, P<0.0001) for males and younger participants. The female had an inverse association with the ALT (β = -0.285, P<0.0001), age and BMI [Table. 5].

Table 5 : Regression **analysis of the** effects **of** the **TyG index** , sex, age, **and** BMI **on** 43

various metabolic and thyroid parameters in subclinical hypothyroid patients Variable Parameters B B SE T R Square Tolerance (VIF)

P Value FT4 **TyG index 0** .035 **0** .025 **0** .033 1.078 **0** 64

.000 0.888 (1.126) 0.281 Gender (Male) -0.031 -0.150 0.005 -6.44 0.021 0.897 (1.115) <0.0001* Age 0.004 0.039 0.002 1.741 0.002 0.976 (1.024) 0.082 BMI -0.001 -0.010 0.002 -0.439 0.001 0.934 (1.071)) 0.660 T3 TyG index -1.993 -0.313 0.11 -18.001 0.223 0.888 (1.126) <0.0001* Gender -0.471 -0.501 0.016 -29.00 0.361 0.897 (1.115) <0.0001* Age -0.012 -0.028 0.007 -1.675 0.000 0.976 (1.024) 0.094 BMI -0.021 -0.052 0.007 -3.098 0.036 0.934 (1.071)) 0.002* TSH TyG index -0.195 -0.035 0.124 -1.576 0.004 0.888 (1.126) 0.115 Gender 0.255 0.314 0.018 14.06 0.093 0.897 (1.115) <0.0001* Age 0.019 0.048 0.008 2.264 0.001 0.976 (1.024) 0.024 BMI 0.007 0.021 0.007 0.949 0.004 0.934 (1.071)) 0.343 HBA1C TyG index 1.410 0.198 0.158 8.917 0.048 0.888 (1.126) <0.0001* Gender -0.102 -0.097 0.023 -4.377 0.000 0.897 (1.115) <0.0001* Age 0.016 0.032 0.010 1.525 0.000 0.976 (1.024) 0.127 BMI 0.118 0.267 0.010 12.358 0.081 0.934 (1.071)) <0.0001* LDL TyG index 92.675 0.234 8.842 10.482 0.066 0.888 (1.126) <0.0001* Gender 5.830 0.100 1.298 4.492 0.032 0.897 (1.115) <0.0001* Age -4.951 -0.179 0.587 -8.439 0.031 0.976 (1.024) <0.0001* BMI -0.132 -0.005 0.535 -0.247 0.006 0.934 (1.071)) 0.805 HDL TyG index -44.482 -0.221 4.341 -10.247 0.023 0.888 (1.126) <0.0001* Gender 9.130 0.308 0.637 14.329 0.043

0.897 (1.115) <0.0001* Age 2.968 0.212 0.288 10.305 0.043 0.976 (1.024) <0.0001* BMI -1.798 -0.144 0.263 -6.842 0.026 0.934 (1.071)) <0.0001* Cholesterol TyG index 193.147 0.273 15.731 12.278 0.094 0.888 (1.126) <0.0001* Gender 8.975 0.086 2.309 3.887 0.033 0.897 (1.115) <0.0001* Age -4.442 -0.090 1.044 -4.256 0.009 0.976 (1.024) <0.0001* BMI 2.563 0.058 0.952 2.691 0.018 0.934 (1.071)) 0.007* ALT TyG index -0.091 0.00 8.917 -0.010 0.003 0.888 (1.126) 0.992 Gender -16.788 -0.285 1.309 -12.827 0.063 0.897 (1.115) <0.0001* Age -2.354 -0.085 0.592 -3.978 0.009 0.976 (1.024) <0.0001* BMI 4.454 0.180 0.540 8.252 0.021 0.934 (1.071)) <0.0001* ALP TyG index -3.719 -0.004 20.172 -0.184 0.000 0.888 (1.126) 0.854 Gender 22.322 0.173 2.961 7.539 0.024 0.897 (1.115) <0.0001* Age -6.024 -0.099 1.338 -4.500 0.008 0.976 (1.024) <0.0001* BMI -7.051 -0.130 1.221 -5.775 0.008 0.934 (1.071)) <0.0001* Note: *

**A P value less than 0.05 was considered statistically significant**	2

. 272 LSTM Model predictions for TyG index, TSH, T3, and T4 Levels The LSTM model performs well because the predicted values of the TyG index are near the actual values in most of the range. There are minor inconsistencies causing slight inaccuracies in the observed values compared with the expected values. The LSTM model demonstrated high predictive performance for TyG index levels, as the mean squared error (MSE) was low: 0.00034, which is an indicator of a low level of deviation between the predicted and actual values. The changes might also be explained by the fluctuations and differences in the data and shortcomings of the model in the ability to accommodate sudden spikes in the TyG index [Figure 1]. The correlation

**between the predicted** value **and** the **actual value** fits well, **and the**	1

red line becomes closer to the blue line within the samples. The LSTM model is also very precise in predicting the T3 levels since the value of the MSE measure is quite low, indicating that the difference between the actual and approximated numbers is not large. The challenge with modelling this is that some abrupt changes in the scales, such as those of the shaking areas, were impossible to model, although the output of the data project was relatively contending compared with the actual quantity of T3. However, the model sensitivity, in general, in representing the concentration level of T3, indicates that it may be used in its capacity to reflect the degree of unstable thyroid hormones in patients with SCH [Figure 2]. There has been proximity between the two lines, and the predictions have been quite on par with the actual values. MSE=0.0113 is evidence that the LSTM model can predict FT4 levels quite accurately. The model can be useful in estimating most of the variants of FT4 and does not extend exactly to the fast-varying part, particularly in the spiky shoulders. Nevertheless, the overall findings of this model indicate that identifying patients with thyroid-related medical conditions might be beneficial with respect to the assessment of the level of FT4 [Figure 3]. The blue trend represents the observed values of the TSH concentrations in the data, whereas the red trend depicts the predicted values of the TSH concentrations in the data modelled via the LSTM. The 28 results are anticipated based on the general dynamics of the real data; however, there are some inconsistent figures in areas with drastic fluctuations. The findings provided by the LSTM model show that the

prediction is average since the MSE is equal to 0.0426. This model functions well in reproducing the overall trend shifts in the TSH concentration but cannot be applied to reproducing instances where an abrupt decrease or increase in the TSH concentration occurs. Compared with previous histories of other hormones (T3, FT4), a relatively large-scale MSE suggests that further tuning is needed to refine the model to predict TSH in patients with thyroid-related disorders [Figure 4]. Figure 1: LSTM model prediction for the TyG index: A comparison of the actual (blue line) and LSTM-predicted (red line) values of the TyG index demonstrated high predictive accuracy (MSE = 0.00034) in subclinical hypothyroid patients. The vertical x-axis represents the standardized TyG index values, and the horizontal axis represents a sequential index of the data samples on the x-axis (not actual time). A subgroup of 400 samples is represented to help make it clear, which enables one to capture the overall tendency and all minor differences in the TyG 29 index. The small differences between the actual and predicted values reveal that the model indeed can track this relevant metabolic index. Figure 2: LSTM model prediction for T3 levels: The cross-validation results based on time recorded high predictability between real (blue line) and LSTM-predicted (red line) T3 hormone levels in patients with subclinical hypothyroidism (MSE = 0.0015). Among the peculiarities, it is necessary to say that the model can help identify not only a gradual trend but also the process that suggests a sharp alteration in T3 levels, which confirms its effectiveness as a monitoring mechanism of thyroid function. Figure 3: LSTM model prediction for T4 levels. The comparison of the actual (blue line) and LSTM-predicted (red line) T4 hormone levels of subclinical hypothyroid patients indicated that its performance was good (MSE = 0.0113). As depicted by the visualization, the model can capture both long-term changes and short-term changes in the level of T4. Figure 4: Results of the TSH level model. The last model yields a moderate prediction (MSE = 0.0426) because of the complexity of the dynamics of the TSH. The x-axis indicates a sequential sequence of data samples (not actual time), and the y-axis indicates the TSH concentration expressed in mU/L. A small sample of 400 is shown to present significant trends and patterns within the data to provide clarity. Among the notable characteristics are the specificities of the model to follow the general trends in TSH levels yet show some deviations in response to a quick change. Discussion

The correlation between the TyG index and thyroid function **in** SCH **patients** is gaining more **and** 7

more attention in metabolic and endocrine research. Subclinical hypothyroidism (SCH) with elevated TSH but normal FT4 concentration is frequently accompanied with defects in metabolic parameters, such as dyslipidaemia and IR, which are essential elements of the metabolic syndrome [24]. This study findings highlight clear sex- and age-specific differences. Females exhibited higher FPG, cholesterol, HDL, and TSH levels, while males demonstrated higher T3, liver enzymes, and hematological counts. These differences can be explained by hormonal influences: estrogen enhances lipid metabolism and increases thyroid-binding proteins [25], whereas testosterone stimulates erythropoiesis and increases basal metabolic activity [26]. Age-related variations were also evident, with middle-aged individuals showing higher FPG and HbA1c, and triglyceride levels rising with advancing age. These results align with prior reports linking thyroid–metabolic interactions to sex and age [25, 27]. The triglyceride-glucose index (TyG) obtained from FPG and TG is an established surrogate marker for IR [28].

In this study , TyG **was found to be significantly** negatively **associated with** T3 **levels** 41

and significantly

positively associated with HbA1c, **LDL** and total **cholesterol levels** 73

. These results indicate that SCH plays a greater role in lipid disturbance than glycemic imbalance, which again, is in agreement with reports that hypothyroidism increases TG by decreased lipoprotein lipase activity [29, 30]. Conversely, hyperthyroidism reduces TG by accelerating lipid clearance [29, 30]. Moreover,

the findings of this study are consistent with other **studies** 4

that demonstrated TyG is correlated with thyroid function even in euthyroid adults, in whom

low-normal thyroid function is **a** marker **of increased** 65

metabolic risk [31].

Data from the Korean National Health and 33 **Nutrition Examination Survey** 2

confirmed that TSH and FT4 predict TyG levels [31, 32]. However,

other studies found no significant **association between thyroid** dysfunction **and** 58

glucose control, suggesting that lipid metabolism may be the dominant driver of TyG elevations in SCH [27, 33, 34]. The metabolic abnormalities observed—elevated TG, LDL, and HbA1c—demonstrate that SCH is not a benign condition. Even in its subclinical form, hypothyroidism has been associated with insulin resistance and impaired glucose utilization [35, 36]. This highlights

the TyG index as a simple, non-invasive tool for 14

early identification of cardiometabolic risk in SCH. Incorporating TyG into routine screening may help detect high-risk patients earlier, enabling interventions such as lifestyle changes or levothyroxine therapy, which has been shown to improve lipid profiles in some cases [37]. In addition to TyG, our study demonstrated associations between thyroid function and hematological as well as hepatic parameters. Elevated liver enzymes in SCH patients may reflect

non-alcoholic fatty liver disease, which is commonly **linked to** insulin resistance **and** 2

dyslipidemia [38]. This supports previous evidence that thyroid hormones significantly influence lipid metabolism and liver activity [39]. Furthermore, BMI, body composition, and obesity are closely intertwined with thyroid function, reinforcing the role of SCH as a contributor to metabolic syndrome [40, 41].

Strengths and Limitations The strengths of this study include its large sample size and **the use of** 28

both regression analysis and LSTM, which confirmed the associations with high accuracy. However, limitations must be acknowledged: the cross-sectional design precludes causality, convenience sampling and gender imbalance may limit generalizability, and reliance on self-reported histories risks including undiagnosed metabolic conditions [24]. The study relied on self-reported medical 34 history and clinical records as exclusion criteria, which may have led to

the inclusion of participants **with undiagnosed diabetes or** dyslipidemia, potentially influencing **the** 17

results. Conclusion: This study

demonstrated that the TyG index is significantly **associated with** thyroid function parameters **in** subclinical hypothyroid **patients** 44

, showing a negative relationship with T3 and positive associations with HbA1c and lipid markers. These findings indicate that the TyG index reflects thyroid-related metabolic alterations and

may serve as a practical **biomarker for assessing** thyroid dysfunction **risk** 63

. Clinically, its application could support earlier identification of patients at risk of progression and guide timely management. Further longitudinal

studies are needed to validate these associations **and** 66

establish population-specific reference values. By highlighting the role

of the TyG index as a simple and cost- **effective marker for the** early detection **of** 10

metabolic derangements in individuals with subclinical hypothyroidism, this study contributes to broader efforts to improve health equity, prevent chronic disease progression, and promote sustainable well-being in diverse populations. References 1. Li M, Chi X, Wang Y, Setrerrahmane S, Xie W, Xu H: Trends in insulin resistance: insights into mechanisms and therapeutic strategy. Signal Transduction and Targeted Therapy 2022, 7(1):216. 2. Park SY, Gautier JF, Chon S: Assessment of Insulin Secretion and Insulin Resistance in Human. Diabetes Metab J 2021, 45(5):641-654. 3. Fasipe OJ, Ayoade OG, Enikuomehin AC, Falade CO: Evaluating antiretroviral therapy–induced insulin resistance syndrome using the homeostasis model assessment method: an important global clarion call for concern among people living with HIV-disease. RPS Pharmacy and Pharmacology Reports 2024, 3(3):rqae019. 4. Bazyar H, Zare Javid A, Masoudi MR, Haidari F, Heidari Z, Hajializadeh S, Aghamohammadi V, Vajdi M: Assessing the predictive value of insulin resistance indices for metabolic syndrome risk in type 2 diabetes mellitus patients. Scientific Reports 2024, 14(1):8917. 5. Cho YK, Han KD, Kim HS, Jung CH, Park JY, Lee WJ: Triglyceride-Glucose Index Is a Useful Marker for Predicting Future Cardiovascular Disease and Mortality in Young Korean Adults: A Nationwide Population-Based Cohort Study. J Lipid Atheroscler 2022, 11(2):178-186. 6. Li HF, Miao X, Li Y: The Triglyceride Glucose (TyG) Index as a Sensible Marker for Identifying Insulin Resistance and Predicting Diabetic Kidney Disease. Med Sci Monit 2023, 29:e939482. 7. Mullur R, Liu YY, Brent GA: Thyroid hormone regulation of metabolism. Physiol Rev 2014, 94(2):355-382. 8. Eom YS, Wilson JR, Bernet VJ: Links between Thyroid Disorders and Glucose Homeostasis. Diabetes Metab J 2022, 46(2):239-256. 9. Biondi B, Kahaly GJ, Robertson RP: Thyroid Dysfunction and Diabetes Mellitus: Two Closely Associated Disorders. Endocr Rev 2019, 40(3):789-824. 10. Grice A: Subclinical hypothyroidism. InnovAiT 2019, 12(3):131-135. 11. Pearce SH, Brabant G, Duntas LH, Monzani F, Peeters RP, Razvi S, Wemeau J-L: 2013 ETA guideline: management of subclinical hypothyroidism. European thyroid journal 2013, 2(4):215-228. 12. Garber JR, Cobin RH, Gharib H, Hennessey JV, Klein I, Mechanick JI, Pessah-Pollack R, Singer PA, Woeber KA: Clinical practice guidelines for hypothyroidism in adults: cosponsored by the American Association of Clinical Endocrinologists and the American Thyroid Association. Endocr Pract 2012, 18(6):988-1028. 13. Ma CG, Shim YS: Association of Thyroid-Stimulating Hormone and Thyroid Hormones with Cardiometabolic Risk Factors in Euthyroid Children and Adolescents Aged 10-18 Years: A Population-Based

Study. Sci Rep 2019, 9(1):15476. 14. Shin JA, Mo EY, Kim ES, Moon SD, Han JH: Association between Lower Normal Free Thyroxine Concentrations and Obesity Phenotype in Healthy Euthyroid Subjects. Int J Endocrinol 2014, 2014:104318. 15. Canaris GJ, Manowitz NR, Mayor G, Ridgway EC: The Colorado thyroid disease prevalence study. Arch Intern Med 2000, 160(4):526-534. 16. Hollowell JG, Staehling NW, Flanders WD, Hannon WH, Gunter EW, Spencer CA, Braverman LE: Serum TSH, T4, and thyroid antibodies in the United States population (1988 to 1994): National Health and Nutrition Examination Survey (NHANES III). The Journal of Clinical Endocrinology & Metabolism 2002, 87(2):489-499. 17. Emerson CH: Diagnosis and treatment of hypothyroidism: rules, longstanding exceptions, and the emerging entity of thyroid hormone receptor alpha resistance. In., vol. 22: Mary Ann Liebert, Inc. 140 Huguenot Street, 3rd Floor New Rochelle, NY 10801 USA; 2012: 1197-1199. 18. Biondi B, Cooper DS: The clinical significance of subclinical thyroid dysfunction. Endocr Rev 2008, 29(1):76-131. 19. He H, Pan L, Cui Z, Sun J, Yu C, Cao Y, Wang Y, Shan G: Smoking Prevalence, Patterns, and Cessation Among Adults in Hebei Province, Central China: Implications From China National Health Survey (CNHS). Front Public Health 2020, 8:177. 20. Zierle-Ghosh A, Jan A: Physiology, Body Mass Index. In: StatPearls. edn. Treasure Island (FL): StatPearls Publishing Copyright © 2025, StatPearls Publishing LLC.; 2025. 21. Liu C, Liang D: The association between the triglyceride-glucose index and the risk of cardiovascular disease in US population aged ≤ 65 years with prediabetes or diabetes: a population-based study. Cardiovasc Diabetol 2024, 23(1):168. 22. Simental-Mendía LE, Rodríguez-Morán M, Guerrero-Romero F: The product of fasting glucose and triglycerides as surrogate for identifying insulin resistance in apparently healthy subjects. Metab Syndr Relat Disord 2008, 6(4):299-304. 23. Simental-Mendía LE, Rodríguez-Morán M, Guerrero-Romero F: The product of fasting glucose and triglycerides as surrogate for identifying insulin resistance in apparently healthy subjects. Metabolic syndrome and related disorders 2008, 6(4):299-304. 24. Alsulami SS, Baig M, Albeladi AH, Alyoubi SB, Alsubaie SA, Albeladi SA, Ghamri KA, Alraiqi AMS, Alyoubi SM, Almutairi WA: Correlation between Subclinical Hypothyroidism and Metabolic Syndrome: A Retrospective Study. Saudi J Med Med Sci 2023, 11(3):250-256. 25. Lauretta R, Sansone M, Sansone A, Romanelli F, Appetecchia M: Gender in Endocrine Diseases: Role of Sex Gonadal Hormones. Int J Endocrinol 2018, 2018:4847376. 26. Li X, Meng Z, Tan J, Liu M, Jia Q, Zhang G, He Y, Zhang Q, Liu L, Song K et al: Gender impact on the correlation between thyroid function and serum lipids in patients with differentiated thyroid cancer. Exp Ther Med 2016, 12(5):2873-2880. 27. Wang C-Y, Chang T-C, Chen M-F: Associations between subclinical thyroid disease and metabolic syndrome. Endocr J 2012, 59(10):911-917. 28. Lee DY, Lee ES, Kim JH, Park SE, Park CY, Oh KW, Park SW, Rhee EJ, Lee WY: Predictive Value of Triglyceride Glucose Index for the Risk of Incident Diabetes: A 4-Year Retrospective Longitudinal Study. PLoS One 2016, 11(9):e0163465. 29. Hashim AM, Humadi AT: Hypothyroidism, Hyperthyroidism and its Relationship with Lipid Profile in Thyroid Dysfunction Patients. Journal of University of Babylon for Pure and Applied Sciences 2023:122-133. 30. Shin KA, Kim EJ: Association between thyroid hormone and components of metabolic syndrome in euthyroid Korean adults: A population-based study. Medicine (Baltimore) 2021, 100(51):e28409. 31. Choi W, Park JY, Hong AR, Yoon JH, Kim HK, Kang HC: Association between triglyceride-glucose index and thyroid function in euthyroid adults: The Korea National Health and Nutritional Examination Survey 2015. PLoS One 2021, 16(7):e0254630. 32. Choi YM, Kim MK, Kwak MK, Kim D, Hong EG: Association between thyroid hormones and insulin resistance indices based on the Korean National Health and Nutrition Examination Survey. Sci Rep 2021, 11(1):21738. 33. Eom YS, Wilson JR, Bernet VJ: Links between thyroid disorders and glucose homeostasis. Diabetes Metab J 2022, 46(2):239-256. 34. Sakyi SA, Ameyaw B, Laing EF, Anthony R, Ephraim RKD, Effah A, Kwayie AA, Senu E, Anto EO, Acheampong E: Thyroid dysfunction and glycaemic control among Type 2

diabetes mellitus patients in Ghana: A comparative cross-sectional study. Endocrinology, diabetes & metabolism 2023, 6(6):e447. 35. Gronich N, Deftereos SN, Lavi I, Persidis AS, Abernethy DR, Rennert G: Hypothyroidism Is a Risk Factor for New-Onset Diabetes: A Cohort Study. Diabetes Care 2015, 38(9):1657-1664. 36. Wang CY, Chang TC, Chen MF: Associations between subclinical thyroid disease and metabolic syndrome. Endocr J 2012, 59(10):911-917. 37. Razvi S, Weaver JU, Butler TJ, Pearce SHS: Levothyroxine Treatment of Subclinical Hypothyroidism, Fatal and Nonfatal Cardiovascular Events, and Mortality. Archives of Internal Medicine 2012, 172(10):811-817. 38. Xie Y, Wang Z, Chen Z: Analysis of Subclinical Thyroid Dysfunction and Metabolic Abnormality in 28568 Healthy People. Int J Endocrinol 2023, 2023:5216945. 39. Wang L, Chen T, Yu J, Yuan H, Deng X, Zhao Z: Clinical Associations of Thyroid Hormone Levels with the Risk of Atherosclerosis in Euthyroid Type 2 Diabetic Patients in Central China. Int J Endocrinol 2020, 2020:2172781. 40. Yin J, Wang C, Shao Q, Qu D, Song Z, Shan P, Zhang T, Xu J, Liang Q, Zhang S et al: Relationship between the Prevalence of Thyroid Nodules and Metabolic Syndrome in the Iodine-Adequate Area of Hangzhou, China: A Cross-Sectional and Cohort Study. Int J Endocrinol 2014, 2014:675796. 41. Han C, He X, Xia X, Li Y, Shi X, Shan Z, Teng W: Subclinical Hypothyroidism and Type 2 Diabetes: A Systematic Review and Meta-Analysis. PLoS One 2015, 10(8):e0135233. 1 2 3 4 5 6 7 8 9 10 11 12 13 14 15 16 17 18 19 20 21 22 23 24 25 26 27 28 29 30 31 32 33 34 35 36 37 38 39 40 41 42 43 44 45 46 47 48 49 50 51 52 53 54 55 56 57 58 59 60 61 62 63 64 65 66 67 68 69 70 71 72 73 74 75 76 77 78 79 80 81 82 83 84 85 86 87 88 89 90 91 92 93 94 95 96 97 98 99 100 101 102 103 104 105 106 107 108 109 110 111 112 113 114 115 116 117 118 119 120 121 122 123 124 125 126 127 128 129 130 131 132 133 134 135 136 137 138 139 140 141 142 143 144 145 146 147 148 149 150 151 152 153 154 155 156 157 158 159 160 161 162 163 164 165 166 167 168 169 170 171 172 173 174 175 176 177 178 179 180 181 182 183 184 185 186 187 188 189 190 191 192 193 194 195 196 198 199 200 201 202 203 204 205 206 207 208 209 210 211 212 213 214 215 221 222 223 224 225 226 227 228 229 230 231 232 233 234 235 236 237 238 239 240 241 242 243 246 247 248 249 250 251 252 253 254 255 256 257 259 260 261 262 263 264 265 266 267 273 274 275 276 277 278 279 280 281 282 283 284 285 286 287 288 289 290 291 292 293 294 295 296 297 298 299 300 301 302 303 304 305 306 307 308 309 310 311 312 313 314 315 316 317 318 319 320 321 322 323 324 325 326 327 328 329 330 331 332 333 334 335 336 337 338 339 340 341 342 343 344 345 346 347 348 349 350 351 352 353 354 355 356 357 358 359 360 361 362 363 364 365 366 367 368 369 370 371 372 373 374 375 376 377 378 379 380 381 382 383 384 385 386 387 388 389 390 391 392 393 394 395 396 397 398 399 400 401 402 403 404 405 406 407 408 409 410 411 412 413 414 415 416 417 418 419 420 421 422 423 424 425 426 427 428 429 430 431 432 433 434 435 436 437 438 439 440 441 442 443 444 445 446 447 448 449 450 451 452 453 454 455 456 457 458 459 460 461 462 463 464 465 466 467 468 469 470 471 472 473 474 475 476 477 478 479 480 481 482 483 484 485 486 487 488 489 490 491 492 493 494 495 496 497 498 499 500 501 502 503 504 505 506 507 508 509 510 511 512 513 514 515 516 517 518 519 520 521 522 523 524 525 526 527 528 529 530 531 532 533 534 535 536 537 538 539 540 541 542 543 544 545 546 547 548 549 550 551 1 3 4 5 6 7 8 9 10 11 12 13 14 15 16 17 18 19 20 21 22 23 24 25 26 27 30 31 32 35 36 37 38

sources:

1	60 words / 1% - Crossref<br>"Abstracts of the 50th EASD Annual Meeting", Diabetologia, 2014

2 41 words / 1% - from 29-Oct-2024 12:00AM
www.science.gov

3 19 words / < 1% match - from 14-Jan-2025 12:00AM
www.frontiersin.org

4 17 words / < 1% match - from 15-Jan-2025 12:00AM
www.frontiersin.org

5 11 words / < 1% match - from 06-Jan-2025 12:00AM
www.frontiersin.org

6 11 words / < 1% match - from 18-Dec-2023 12:00AM
www.frontiersin.org

7 10 words / < 1% match - from 23-Jan-2025 12:00AM
www.frontiersin.org

8 26 words / < 1% match - Internet from 01-Feb-2023 12:00AM
www.researchgate.net

9 9 words / < 1% match - Internet from 18-Feb-2023 12:00AM
www.researchgate.net

10 13 words / < 1% match - from 26-Jul-2025 12:00AM
cardiab.biomedcentral.com

11 9 words / < 1% match - from 01-Jul-2024 12:00AM
cardiab.biomedcentral.com

12 8 words / < 1% match - Internet from 17-Aug-2022 12:00AM
cardiab.biomedcentral.com

13 18 words / < 1% match - from 22-Dec-2024 12:00AM
bmccardiovascdisord.biomedcentral.com

14 10 words / < 1% match - from 05-Sep-2025 12:00AM
bmccardiovascdisord.biomedcentral.com

15 25 words / < 1% match - from 12-Sep-2023 12:00AM
www.amedeolucente.it

16 14 words / < 1% match - from 08-Dec-2023 12:00AM
journals.plos.org

17 8 words / < 1% match - Internet from 14-Sep-2020 12:00AM
journals.plos.org

18 21 words / < 1% match - Internet from 25-Aug-2022 12:00AM
bmcpublichealth.biomedcentral.com

19 20 words / < 1% match - Crossref
"Day 3 - Monday 30 August 2004", European Heart Journal, 09/02/2004

20 12 words / < 1% match - Internet from 10-Nov-2020 12:00AM
academic.oup.com

21 8 words / < 1% match - Internet from 25-Feb-2023 12:00AM
academic.oup.com

22 19 words / < 1% match - from 07-Sep-2024 12:00AM
bmcendocrdisord.biomedcentral.com

23 19 words / < 1% match - from 21-Aug-2023 12:00AM
www.sciencegate.app

24 18 words / < 1% match - from 24-Jul-2024 12:00AM
www.thenews.com.pk

25 17 words / < 1% match - Internet from 08-Jan-2023 12:00AM
www.lalpathlabs.com

26 16 words / < 1% match - Crossref
Chengping Hu, Jianwei Zhang, Jinxing Liu, Yan Liu, Ang Gao, Yong Zhu, Yingxin Zhao. "Discordance between the triglyceride glucose index and fasting plasma glucose or HbA1C in patients with acute coronary syndrome undergoing percutaneous coronary intervention predicts cardiovascular events: a cohort study from China", Cardiovascular Diabetology, 2020

27 16 words / < 1% match - Internet from 25-Nov-2017 12:00AM
repub.eur.nl

28 16 words / < 1% match - from 13-May-2025 12:00AM
www.researchsquare.com

29 15 words / < 1% match - from 02-Dec-2023 12:00AM
assets.researchsquare.com

30 15 words / < 1% match - from 25-Jan-2024 12:00AM
eurjmedres.biomedcentral.com

31 15 words / < 1% match - from 16-Dec-2024 12:00AM
pmc.ncbi.nlm.nih.gov

32 14 words / < 1% match - from 26-May-2025 12:00AM
researchspace.auckland.ac.nz

33 13 words / < 1% match - from 29-Jul-2024 12:00AM
dspace.ewha.ac.kr

34 13 words / < 1% match - Internet from 14-Aug-2020 12:00AM
lipidworld.biomedcentral.com

35 13 words / < 1% match - Internet from 28-Sep-2022 12:00AM
www.scielo.br

36 12 words / < 1% match - Crossref
"Sunday, 2 September 2007", European Heart Journal, 09/02/2007

37 12 words / < 1% match - from 17-Apr-2024 12:00AM
jgmds.org.pk

38 12 words / < 1% match - from 13-Sep-2024 12:00AM
jkimsu.com

39 12 words / < 1% match - Internet from 17-Oct-2022 12:00AM
www.researchprotocols.org

40 11 words / < 1% match - Crossref
Chenyu Zhang, Haoyu Wang, Yongze Li, Xichang Wang et al. "Association between the triglyceride-glucose index and thyroid disorders: a cross-sectional survey and Mendelian randomization analysis", Endocrine, 2024

41 11 words / < 1% match - ProQuest
Joseph, Ray Mond Mark. "Total Testosterone and Asymmetric Dimethylarginine: Association of Demographic, Clinical and Lifestyle Factors with the Severity of Erectile Dysfunction", University of Malaya (Malaysia), 2023

42 11 words / < 1% match - Crossref
Khalid Siddiqui, Shaik Sarfaraz Nawaz, Teena P. George, Satish Kumar David, Assim A Alfadda, Mohamed Rafiullah. "Association of triglyceride-glucose index with diabetic kidney disease in patients with type 2 diabetes", Journal of Diabetes & Metabolic Disorders, 2025

43 10 words / < 1% match - Crossref
Yingqi Shan, Qingyang Liu, Tianshu Gao. "Interaction of triglyceride glucose index and weight-adjusted waist circumference index in the risk of diabetes: from a national cohort study", BMC Endocrine Disorders, 2025

44 10 words / < 1% match - from 04-Sep-2024 12:00AM
bmcmedicine.biomedcentral.com

45 10 words / < 1% match - from 19-Jul-2024 12:00AM
impactfactor.org

46 10 words / < 1% match - Internet from 12-Jan-2023 12:00AM
journals.lww.com

47 10 words / < 1% match - from 15-Apr-2025 12:00AM
pediatrics.jmir.org

48 10 words / < 1% match - Internet from 13-Nov-2021 12:00AM
www.amhsr.org

49 9 words / < 1% match - Crossref
Pronoy Kumer Sarker, Nisat Sultana, Hafizul Islam, Md Samiul Bashir, Md. Sujon Ali. "Thyroid dysfunction and metabolic dysregulation: A cross-sectional study of hormonal and glycemic parameters", Molecular Mechanism Research, 2025

50 9 words / < 1% match - Crossref
Xin Chen, Jinyou Yang, Dan Wang, Jiali Liu, Hang Jin, Yongqing Zhang, Quanyong Xiang. "Impact of triglyceride-glucose index on risk of cardiovascular disease among non-diabetic hypertension patients: a 10-year prospective cohort study", BMC Public Health, 2025

51 9 words / < 1% match - from 05-Jul-2025 12:00AM
bmcgeriatr.biomedcentral.com

52 9 words / < 1% match - Internet from 05-Mar-2022 12:00AM
journals.sums.ac.ir

53 9 words / < 1% match - from 22-Jan-2025 12:00AM
medium.com

54 9 words / < 1% match - from 22-Apr-2025 12:00AM
rua.ua.es

55 9 words / < 1% match - from 14-Jul-2024 12:00AM
www.mdedge9-ma1.mdedge.com

56 8 words / < 1% match - Crossref
"Society of general internal medicine 23rd annual meeting Boston, Massachusetts May 4–6, 2000 abstracts", Journal of General Internal Medicine, 2000

57 8 words / < 1% match - Crossref
Afrin Pathan, Javed Yasin, Charu Sharma, Juma Alkaabi, Adnan Agha. "Assessing thyroid peroxidase antibodies in Emirati medical students: a cross-sectional pilot study", Frontiers in Endocrinology, 2025

58 8 words / < 1% match - Crossref
Ladan Mehran, Atieh Amouzegar, Fereidoun Azizi. "Thyroid disease and the metabolic syndrome", Current Opinion in Endocrinology & Diabetes and Obesity, 2019

59 8 words / < 1% match - from 26-Jun-2023 12:00AM
bcsrj.com

60 8 words / < 1% match - from 24-Oct-2024 12:00AM
www.forumortodontyczne.pl

61 8 words / < 1% match - from 20-Apr-2025 12:00AM
www.ijddc.org

62 8 words / < 1% match - from 27-Dec-2024 12:00AM
www.journal.smdc.edu.pk

63 8 words / < 1% match - from 19-Jun-2025 12:00AM
www.pps.org.pk

64 8 words / < 1% match - Internet from 10-Jun-2019 12:00AM
www.tandfonline.com

65 7 words / < 1% match - Crossref
"Thyroid and Heart", Springer Science and Business Media LLC, 2020

66 7 words / < 1% match - Crossref
Huang Chen Chang, Jun-Peng Chen, Yi-Ming Chen, Wen-Nan Huang Yi-Hsing Chen. "ANTI-C1Q ANTIBODIES AS INDICATORS OF DISEASE ACTIVITY, RENAL INVOLVEMENT, AND NON-SCARRING ALOPECIA IN PATIENTS WITH SLE", The Journal of Rheumatology, 2025

67	7 words / < 1% match - Crossref<br>Hui Cheng, Yanyan Hu, Haoran Zhao, Guowei Zhou, Gaoyuan Wang, Chaoqun Ma, Yan Xu. "Exploring the association between triglyceride-glucose index and thyroid function", European Journal of Medical Research, 2023
68	7 words / < 1% match - Crossref<br>Huimin Cao, Yuying Zhao, Ziyi Chen, Xiaoya Zou et al. "Triglyceride-glucose index predicts cognitive decline and striatal dopamine deficiency in Parkinson disease in two cohorts", npj Parkinson's Disease, 2025
69	7 words / < 1% match - Crossref<br>Kengo Moriyama, Yumi Masuda, Nana Suzuki, Chizumi Yamada, Noriaki Kishimoto, Shinji Takashimizu, Akira Kubo, Yasuhiro Nishizaki. "Estimated Elovl6 and delta-5 desaturase activities might represent potential markers for insulin resistance in Japanese adults", Journal of Diabetes & Metabolic Disorders, 2022
70	7 words / < 1% match - Crossref<br>Sandeep Samethadka Nayak, Dona Kuriyakose, Lakshmi D. Polisetty, Anjali Avinash Patil et al. "Diagnostic and prognostic value of triglyceride glucose index: a comprehensive evaluation of meta-analysis", Cardiovascular Diabetology, 2024
71	7 words / < 1% match - Crossref<br>Wonsuk Choi, Ji Yong Park, A. Ram Hong, Jee Hee Yoon, Hee Kyung Kim, Ho-Cheol Kang. "Association between triglyceride-glucose index and thyroid function in euthyroid adults: The Korea National Health and Nutritional Examination Survey 2015", PLOS ONE, 2021
72	7 words / < 1% match - Crossref Posted Content<br>Yingying Tang, Lei Fan, Xiukun Zhang, Chengyuan Li, Fuman Du. "Effect of Normal Level Endocrine Hormones and Hypothalamic Neuropeptides on Obesity in Women of Childbearing Age", Springer Science and Business Media LLC, 2025
73	6 words / < 1% match - Publications<br>Barry D. Smith, Uma Gupta, B.S. Gupta. "Caffeine and Activation Theory - Effects on Health and Behavior", CRC Press, 2006
74	6 words / < 1% match - Crossref<br>Eunji Mun, Hye Ah Lee, Jung Eun Choi, Rosie Lee, Kyung Hee Kim, Hyesook Park, Hae Soon Kim. "Association between Thyroid Function and Insulin Resistance Indices in Korean Adolescents: Findings from the 2014–2015 Korea National Health and Nutrition Examination Survey", Children, 2024
75	6 words / < 1% match - Crossref<br>James V. Hennessey, Ramon Espaillat. "Reversible morbidity markers in subclinical hypothyroidism", Postgraduate Medicine, 2014
76	6 words / < 1% match - Crossref

Jeffrey R. Garber, Rhoda H. Cobin, Hossein Gharib, James V. Hennessey et al. "Clinical Practice Guidelines for Hypothyroidism in Adults: Cosponsored by the American Association of Clinical Endocrinologists and the American Thyroid Association", Thyroid, 2012

77 6 words / < 1% match - Crossref Posted Content
Jingya Zhao, Xinning Lu, Hui Wang, Yuanqin Li et al. "Associations of the triglyceride–glucose index, triglyceride glucose–body mass index and waist–triglyceride index with mortality in patients with cardiovascular–kidney–metabolic syndrome stages 0-4: evidence from NHANES 1999-2020", Springer Science and Business Media LLC, 2025

78 6 words / < 1% match - Crossref
Song Zhao, Shikai Yu, Chen Chi, Ximin Fan, Jiamin Tang, Hongwei Ji, Jiadela Teliewubai, Yi Zhang, Yawei Xu. "Association between macro- and microvascular damage and the triglyceride glucose index in community-dwelling elderly individuals: the Northern Shanghai Study", Cardiovascular Diabetology, 2019

79 6 words / < 1% match - Crossref
Yu Yan, Dan Wang, Yue Sun, Qiong Ma et al. "Triglyceride-glucose index trajectory and arterial stiffness: results from Hanzhong Adolescent Hypertension Cohort Study", Cardiovascular Diabetology, 2022

80 6 words / < 1% match - Internet
Lirui Zhang, Xin Yan, Wei Zheng, Xianxian Yuan et al. "Assessment of first-trimester insulin resistance indices for gestational diabetes mellitus: a prospective cohort study", Journal of Endocrinological Investigation
